# How much multiple paternity should we expect? A study of birds and contrast with mammals

**DOI:** 10.1002/ece3.11054

**Published:** 2024-03-01

**Authors:** F. Stephen Dobson, Hannah E. Correia, Ash Abebe

**Affiliations:** ^1^ Department of Biological Sciences Auburn University Auburn Alabama USA; ^2^ Unité Mixte de Recherche 7178, Institut Pluridisciplinaire Hubert Curien, Centre National de la Recherche Scientifique Université de Strasbourg Strasbourg France; ^3^ Department of Environmental Health and Engineering Johns Hopkins University Baltimore Maryland USA; ^4^ Department of Mathematics and Statistics Auburn University Auburn Alabama USA

**Keywords:** Bayesian modeling, birds, brood size, litter size, mammals, MCMC sampling, multiple paternity, truncated Poisson distribution

## Abstract

Parentage analyses via molecular markers have revealed multiple paternity within the broods of polytocous species, reshaping our understanding of animal behavior, ecology, and evolution. In a meta‐analysis of multiple paternity in bird and mammal species, we conducted a literature search and found 138 bird and 64 mammal populations with microsatellite DNA paternity results. Bird populations averaged 19.5% multiple paternity and mammals more than twice that level (46.1%). We used a Bayesian approach to construct a null model for how multiple paternity should behave at random among species, under the assumption that all mated males have equal likelihood of siring success, given mean brood size and mean number of sires. We compared the differences between the null model and the actual probabilities of multiple paternity. While a few bird populations fell close to the null model, most did not, averaging 34.0‐percentage points below null model predictions; mammals had an average probability of multiple paternity 13.6‐percentage points below the null model. Differences between bird and mammal species were also subjected to comparative phylogenetic analyses that generally confirmed our analyses that did not adjust for estimated historical relationships. Birds exhibited extremely low probabilities of multiple paternity, not only compared to mammals but also relative to other major animal taxa. The generally low probability of multiple paternity in birds might be produced by a variety of factors, including behaviors that reflect sexual selection (extreme mate guarding or unifocal female choice) and sperm competition (e.g., precedence effects favoring fertilization by early or late matings).

## Graphical abstract

We compared the degree of multiple paternity in species of birds and mammals. Birds average multiple paternity in about 20% of broods, and mammals in 46% of litters. The low rates of multiple paternity in birds might result from strong sexual selection and sperm competition.

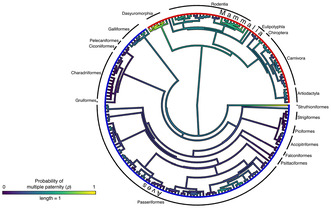

## 1 INTRODUCTION

Natural selection and the evolutionary fitness of trait forms depend on reproductive success, which may often result at least in part from patterns of matings. Mating patterns, in turn, are influenced by the attributes of particular species, such as the variation and extent of existing trait forms, and by the characteristics of the environments in which species occur. Characteristics of social and ecological environments may thus produce fitness differences among trait forms (Endler, 1986). Mating patterns are often closely associated with reproductive success, which, together with inheritance, determines the particular trait forms that are passed on to future generations. Mating patterns and reproductive success, however, are not quite the same thing.

The degree to which reproductive success is reflected in patterns of mating may depend on many social and environmental factors. For example, the reproductive success of females often depends on offspring production and care, but for males, success may depend on the number of females that they mate and inseminate (Emlen & Oring, 1977). Thus, an important aspect of mating for males is siring success, both within and among females. For species that commonly produce a single offspring at each reproductive event, male success is dominated by the number of mates and ultimately the sirings obtained. For polytocous species (i.e., those that produce more than a single offspring at a time), variation in reproductive success among males also depends on the proportions of sired young among their mates. Thus, for these latter species, whether the paternity of broods or litters is single or multiple is an important aspect of reproductive success.

Considerable variation in mating patterns occurs among avian species, yet a majority of species are socially monogamous, polytocous, and pair‐living (Griffith et al., 2002; Lack, 1968). Genetic analyses, however, have revealed that most socially monogamous species, as well as birds in general, have some degree of multiple paternity within their populations (reviews by Biagolini‐Jr et al., 2017; Brouwer & Griffith, 2019; Valcu et al., 2021). These studies used molecular paternity identification techniques to examine whether male social partners of females are also genetic sires of broods of nestlings (Bennett & Owens, 2002; Black, 1996; Ligon, 1999; Westneat et al., 1990). Such studies often focused on the presence of extra‐pair paternity (EPPs), a phenomenon that was frequently present at fairly low levels (e.g., 0–30%; see Figure 2 in Brouwer & Griffith, 2019). At the same time, some of the studies presented results that documented the mean probability of multiple paternity within populations. The occurrence of multiple paternity in these bird populations prompts the question of how much multiple paternity should be expected.

Avian species are excellent subjects for analyzing multiple paternity, primarily due to the interest of ornithologists in explaining why EPPs occur (Brouwer & Griffith, 2019; Valcu et al., 2021). Bird species that are socially monogamous might be expected to exhibit relatively low levels of multiple paternity. But at the same time, birds are highly mobile and often occupy a complex, three‐dimensional environment. This facilitates intersexual contacts with a variety of mating partners, which might lead to high levels of multiple paternity. While many factors might contribute to which males get to mate with a female, such as limits to access and acceptance of copulation by the female, significant and informative variation is also likely to occur in siring success among those males that obtain matings. Quantification of this latter variation requires a null model of the success of mating males.

If inseminations were from a random selection of sperm, with all sperm‐contributing males having the same chance of being selected as sires, then the probability of multiple paternity should depend on the number of potential sires, the number of offspring in a brood, and the probability of siring success. This equal likelihood of siring success assumption is reasonable for setting up a null hypothesis for estimating the expected probability of multiple paternity, allowing us to estimate probabilities of multiple paternity that we should expect if no external factors influenced variation in individual siring success directly from observed numbers of sires and brood or litter sizes (Dobson et al., 2018). Variation in siring success, however, may change under the presence or absence of multiple paternity (e.g., Byers et al., 2004; Garg et al., 2012). The magnitude of deviation in multiple paternity from a scenario of equal siring success provides a meaningful quantification of the differences in reproductive success among populations and species that occur not only due to differences in mating patterns, but perhaps also other features of the species' biology. Thus, such deviations of multiple paternity from a null model should stimulate directions for further study of mating success.

Our purpose in the present study was to test the extent to which observed probabilities of multiple paternity within bird populations deviated from the estimated probability of multiple paternity under an appropriate null model of random fertilizations with equal chances of success among potential sires. To evaluate whether patterns of multiple paternity in birds differ from those of other vertebrate species, we compared results from an analysis of multiple paternity in birds to those of mammals. We chose mammals as a comparative group because mammalian species have the greatest number of populations studied after birds (Avise & Liu, 2011; Correia et al., 2021). Further, broods of birds in the nest and litters of mammals have generally been accurately sampled, and the numbers of offspring in broods of oviparous birds and litters of viviparous mammals are similar.

Null model predictions under random fertilization with equal chance of siring success in mammals indicated that at relatively low litter sizes (viz., those below about 10), the probability of multiple paternity increased with litter size (Abebe et al., 2019; Correia et al., 2021; Dobson et al., 2018). We thus hypothesized that birds should exhibit similar observed patterns of multiple paternity as mammals, since they have similar distributions of brood and litter sizes (Burley & Parker, 1998; Eccard & Wolf, 2009; Parker & Tang‐Martinez, 2004). Results from mammals indicated that species exhibited probabilities of multiple paternity that were on average about 15 percentage points below the expected probabilities of multiple paternity under the null model (Dobson et al., 2018). Thus, we did not expect birds to adhere to the null model either. Comparatively low rates of EPPs that are widespread among birds (Brouwer & Griffith, 2019) suggest that many populations might exhibit relatively low probabilities of multiple paternity in comparison to the predictions of our null model and relative to the pattern found in mammalian populations.

Comparisons of birds and mammals necessarily involve only two groups, and there was little chance that their average rates of multiple paternity would be the same. Our questions were which taxon exhibited the greatest average rate of multiple paternity, which taxon deviated most from our null model, and finally, what the degree of difference was. Thus, our primary focus was on effect sizes. To further elaborate on the differences between birds and mammals, we also applied phylogenetic contrasts. With these analyses, we had two objectives: first, to quantify the degree of historical pattern of multiple paternity and of deviations from our null model within bird and mammal species; second, to evaluate differences between birds and mammals in multiple paternity while removing the influence of history with a comparative phylogenetic analysis. Consistent differences between nonphylogenetic and phylogeny‐adjusted comparisons should increase our confidence in the magnitude of observed effects (Price, 1997).

## 2 METHODS

We searched the Web of Science for studies on birds that reported results on multiple paternity (last searched 20 March 2023). Since ornithologists have mainly been interested in EPPs, especially in socially monogamous species, more studies (451) were revealed by searching “All Databases” for “bird*” and “EPP*” than for other combinations that included “Aves” or “multiple paternit*.” For mammals, we searched for “mammal*” and “multiple paternit*,” generating 425 studies. We reviewed these studies, and discarded 225 on birds and 367 on mammals that did not fit our criteria (see below). In addition to these studies, we examined studies used by two extensive review papers: Brouwer and Griffith (2019) for birds and Avise and Liu (2011) for mammals. For the species in this final pool, there were a few cases (3 in birds and 8 in mammals) where estimates of mean brood or litter size were lacking, and we found and used estimates from other studies (in birds, by the same authors on the same study sites; alternative authors and sites for mammals). The body of literature generated 202 species and 247 populations of birds and 61 species and 64 populations of mammals that were used in the meta‐analyses (Appendix A, B).

Every study included in our meta‐analysis needed to explicitly report or have sufficient information to calculate the following statistics: mean brood or litter size, mean number of sires per brood or litter, the number of broods or litters studied, and the proportion of broods or litters that exhibited multiple paternity. Where these values were reported by authors, we recorded them. Where other information was provided, we calculated the necessary values. For mean brood or litter size, we divided the total number of young by the number of broods or litters. For the mean number of sires, we summed the number of sires among broods (e.g., broods with 1 sire, 2 sires, 3 sires, etc.) and divided by the number of broods or litters. For the proportion of broods or litters that exhibited multiple paternity, we divided the number of broods or litters with more than one sire by the number of broods or litters. Cases of conspecific nest parasitism by females (viz., “egg dumping”) were not included. Some of the bird and mammal species were studied at more than one place or time, and these populations were analyzed separately for empirical comparisons and combined for estimation of the null model.

### 2.1 Null model of multiple paternity

Brommer et al. (2007) reviewed the use of null models that examine EPPs in broods of birds and emphasized that such models should have clear criteria of randomness and explicit assumptions. The reviewed models generally focused on the probability of extra‐pair copulations, that is, copulations by a female with males other than her socially paired mate. Thus, the modeling was based on the probability that the young in a brood had a sire other than the social male partner of the male–female pair (Brommer et al., 2007, 2010; Cramer, 2021; Cramer, Greig, et al., 2020; Cramer, Kaiser, et al., 2020). This focus on the likelihood of EPPs did not explicitly incorporate the number of sires in a brood, different from our null model formulation of multiple paternity, which is based on the probability of the number of sires being greater than one in a brood (Dobson et al., 2018). Because some broods might contain many sires, rarely up to the number of offspring in a brood, our null model was more appropriate than null models reviewed by Brommer et al. (2007) for the empiricism that we analyzed; that is, with respect to the probability of multiple paternity within species.

We used our previously developed null model to obtain the expected probability of multiple sires under random fertilization with an equal chance of siring success for mating males (Abebe et al., 2019; Correia et al., 2021; Dobson et al., 2018). We used an equation for multiple paternity defined as the probability of more than one sire occurring in a brood or litter, then estimated the probability of successful fertilization (i.e., siring success) for all mating males of a species given observed proportions of multiple paternity, litter sizes, and numbers of sires reported in the literature (Appendix 1: Section S1; Correia et al., 2021; Dobson et al., 2018). We then calculated a probability of multiple paternity for litters or broods under the null model constraint of no variation in siring success among potential sires, using the estimated probability of siring success, average litter sizes, and average number of sires for each species (Abebe et al., 2019; Dobson et al., 2018). Estimation of the probability of siring success was conducted using a Bayesian model with Markov chain Monte Carlo (MCMC) sampling (Appendix 1: Section S1) with the *rjags* package in R and JAGS version 4.3.0 (Plummer, 2017, 2022; R Core Team, 2022). We compared the expected probability of multiple paternity under the null model ($p_{B}$) with the observed probability of multiple paternity (p) for each species and quantified the deviation from the prediction of the null model as $p_{B}-p$.

### 2.2 Examination of multiple paternity in birds

The molecular techniques used to identify multiple paternity in birds had some special properties. For birds, DNA fingerprinting techniques were applied early on (viz., the 1990s) and were developed well before molecular paternity studies of mammalian species. Early avian studies often extracted nondestructive blood samples, a technique that used the nucleated blood cells of birds for application in the laboratory. In comparison, mammal blood cells are not nucleated, so mammalian studies awaited the advent of nonblood tissue sampling and microsatellite DNA analyses. Studies of birds have thus applied both types of laboratory analyses in paternity studies, but the use of single‐nucleotide polymorphisms (SNPs) may replace both in future (Flanagan & Jones, 2019).

In any single study and using DNA fingerprinting or microsatellite DNA techniques, it might be easier to identify cases of zero multiple paternity or underestimate the number of sires than cases with multiple sires in broods or litters, due to uncertain sire assignments. For birds, we had sufficient samples of each of the two molecular techniques that have been used to estimate multiple paternity—DNA fingerprinting and microsatellite DNA analyses—to test whether they differed. Thus, we compared the odds of detecting probabilities of multiple paternity equal to zero between techniques while controlling for brood size using the Cochran–Mantel–Haenszel (CMH) test (Agresti, 2002; see Appendix 1: Section S2). We also compared the proportions of multiple paternity in studies of bird populations that used DNA fingerprint and microsatellite DNA techniques using a clustered Wilcoxon rank sum test (Datta & Satten, 2005; see Appendix 1: Section S2). We found DNA fingerprinting studies to produce significantly lower estimates of multiple paternity than later studies that used microsatellite DNA, perhaps because the former were based on an unknown number of alleles per locus (Chambers et al., 2014; see Appendix 1: Section S2 for complete analyses and results). Because of this, and because we compared our results for birds to similar results for mammals that only applied microsatellite DNA techniques, we restricted the remainder of the analyses to studies that used microsatellite DNA methods.

The CMH and clustered Wilcoxon rank sum tests were also used to compare bird species that were described as primarily “socially monogamous,” or behaviorally “bonded” or “paired” male and female mates, to bird species with other types of behavioral mating systems (viz., polygyny, polyandry, polygynandry, and variable cases where two or more types of mating combinations were common). The CMH test is a nonparametric procedure designed for testing independence in two binary datasets in the presence of a stratified possible confounder. For birds, the CMH test was applied to determine whether behavioral mating systems (“socially monogamous” vs. “other”) differed in the presence of multiple paternity while controlling for brood size. Average brood size was rounded into integer clusters (e.g., broods with an average number of offspring between 1.5 and 2.5 were gathered into a single cluster representing average brood sizes around 2, while those between 2.5 and 3.5 were put into another cluster representing average brood sizes near 3, and so forth for the entire range of brood sizes in birds and mammals). Sample sizes for mean brood sizes less than 1.5 (*n* = 5 analyses using fingerprinting, *n* = 4 analyses using microsatellites), and for mean brood sizes greater than 7.0 (*n* = 4 analyses using fingerprinting, *n* = 7 analyses using microsatellites) were low. Therefore, mean brood sizes of at most 2.5 were grouped into a single cluster, and mean brood sizes greater than 6.5 were also grouped into a single cluster. A 2 × 2 × 6 contingency table was thus constructed with the counts of analyses that had zero and nonzero multiple paternity for each of the two methods across six clusters of brood sizes. The conditional log odds ratios (LOR) were calculated for each brood size cluster, and the common odds ratio (COR) and common log odds ratios (CLOR) were calculated overall, corresponding to the test of independence using the CMH procedure. The significance of the LOR and CLOR was calculated using an exact permutation test.

The two bird mating system categories (“socially monogamous” vs. “other”) were tested for a significant difference in the proportions of multiple paternity and their deviations from the null model using a clustered Wilcoxon rank sum test (Datta & Satten, 2005), where brood size was clustered as for the CMH test. While the CMH test examines binary data, the Wilcoxon rank sum test for clustered data examines the extent of difference between two categories of ordinal or metric data while accounting for a clustered confounder and without making any distributional assumptions. This makes the clustered Wilcoxon test an ideal method for observed probabilities of multiple paternity and their deviations from the null model, where we expected nonlinear associations with brood size. An approximation of an exact permutation test using 2000 random permutations was used to calculate the significance of the clustered Wilcoxon rank sum test. We hypothesized that species with socially monogamous mating systems would exhibit lower probabilities of multiple paternity than those with more variable mating systems.

### 2.3 Comparison of birds to mammals

Before comparing multiple paternity in birds to that of mammals, we determined the consistency of multiple observations of average brood/litter size and multiple paternity within each taxa for species with more than one population studied using Krippendorff's alpha (α), which varies between 0 (inconsistent) and 1 (perfectly consistent; Krippendorff, 1980). Krippendorff's alpha is a function of the proportion of observed inconsistency to the inconsistency expected from randomly assigned values to variables and thus serves as an index of the consistency of independent studies to measure the same variables. The 95% confidence interval, estimated through bootstrapping, was also calculated for each Krippendorff's alpha.

A comparison of multiple paternity in birds to that of mammalian species tested both the probability of zero multiple paternity and the hypothesis that the probability of multiple paternity in bird populations might be especially low. Under the assumption of equal likelihood of siring success by mating males, the predicted increase in multiple paternity with brood or litter size is nonlinear (Abebe et al., 2019; Correia et al., 2021; Dobson et al., 2018). Thus, a CMH test (Agresti, 2002) examined whether birds or mammals were more likely to exhibit zero values of the observed probability of multiple paternity. Statistical comparisons of observed probabilities of multiple paternity and their deviations from our null models for birds and mammals were made using the clustered Wilcoxon rank sum test (Datta & Satten, 2005), where brood/litter size was clustered as for the tests between the alternative DNA‐based techniques of paternity identification in birds.

Average brood/litter sizes, average number of sires, and number of broods sampled were compared for populations of birds and mammals analyzed with microsatellite DNA techniques using t‐tests. These tests were made on empirical data from field and laboratory studies, and thus formed a supportive background to our tests that used estimates from our null model.

### 2.4 Phylogenetic contrasts

**FIGURE 1:**
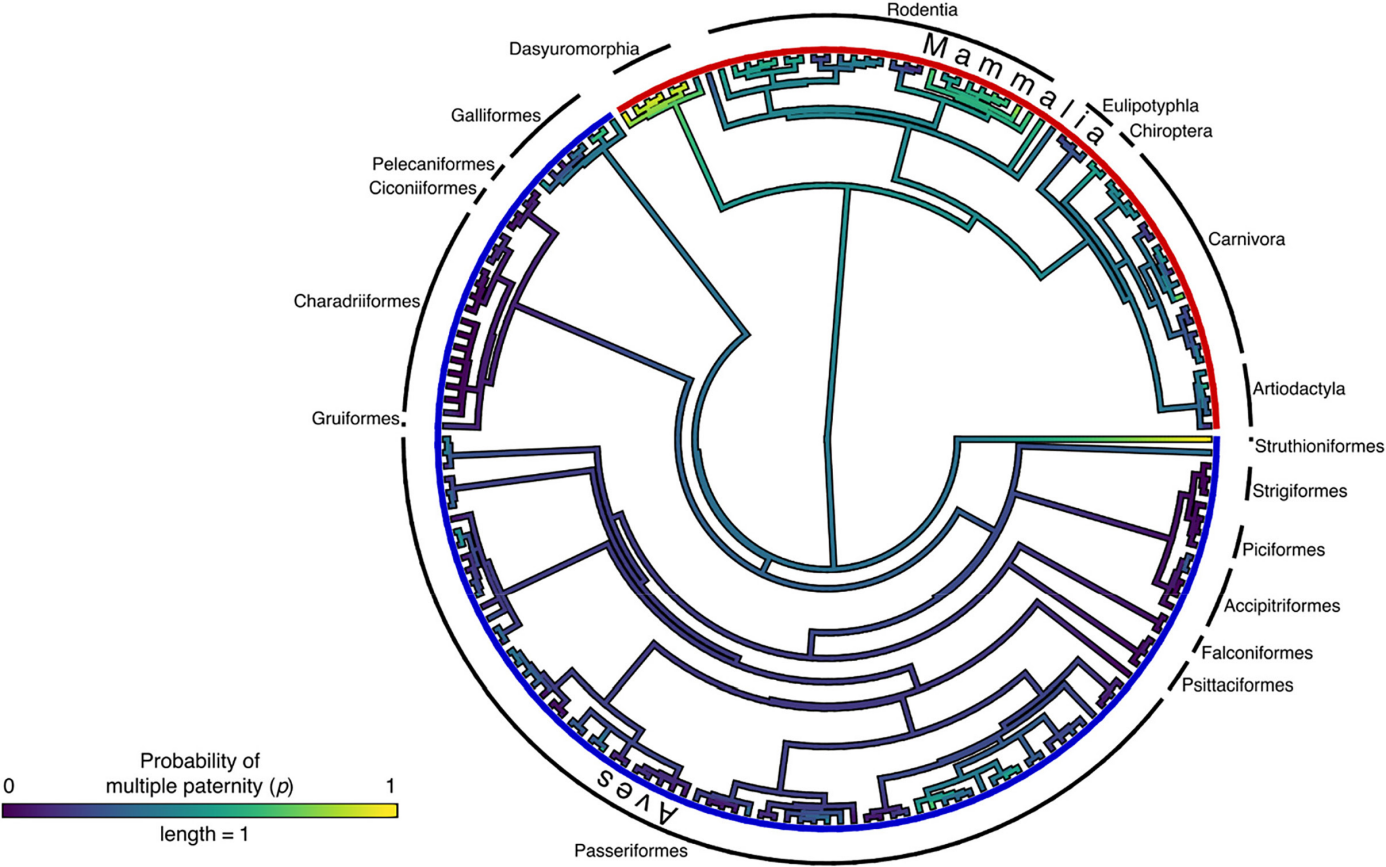
Probability of multiple paternity (p) across the combined phylogeny including avian and mammalian species.

We generated consensus trees for birds and mammals (Jetz et al., 2012; Upham et al., 2019), grafted those trees onto the phylogenetic tree for Amniota to create a single combined tree (Kumar et al., 2022), and compared probabilities of multiple paternity and deviations from the null model between birds and mammals and between socially monogamous and nonsocially monogamous species within and between these taxa, using Bayesian phylogenetic mixed models to account for associations with phylogenetic patterns (as represented by a molecular‐based phylogenetic tree, Figure 1; Appendix 1: Section S3). To provide context, we also conducted a phylogenetic comparative analysis of mean body mass for the bird and mammal species, a variable that exhibits a strong historical pattern (e.g., Dobson et al., 2021; Dobson & Oli, 2007). Body mass data were obtained from PanTHERIA (Jones et al., 2009), from the Animal Diversity Web (Myers et al., 2023), from *Mammalian Species* (https://www.mammalsociety.org/publications/mammalian‐species), and from Birds of the World (Billerman et al., 2022), and Bayesian phylogenetic models were fit for body mass of birds and mammals (Appendix 1: Section S3). We also calculated the heritability of the probability of multiple paternity, the deviations of multiple paternity from the null model, and body mass as the proportion of variation attributable to additive genetic variance across species within each specified taxon (Ives & Helmus, 2011; Nakagawa et al., 2017; Appendix 1: Section S3).

## 3 RESULTS

### 3.1 Analyses of birds

**FIGURE 2:**
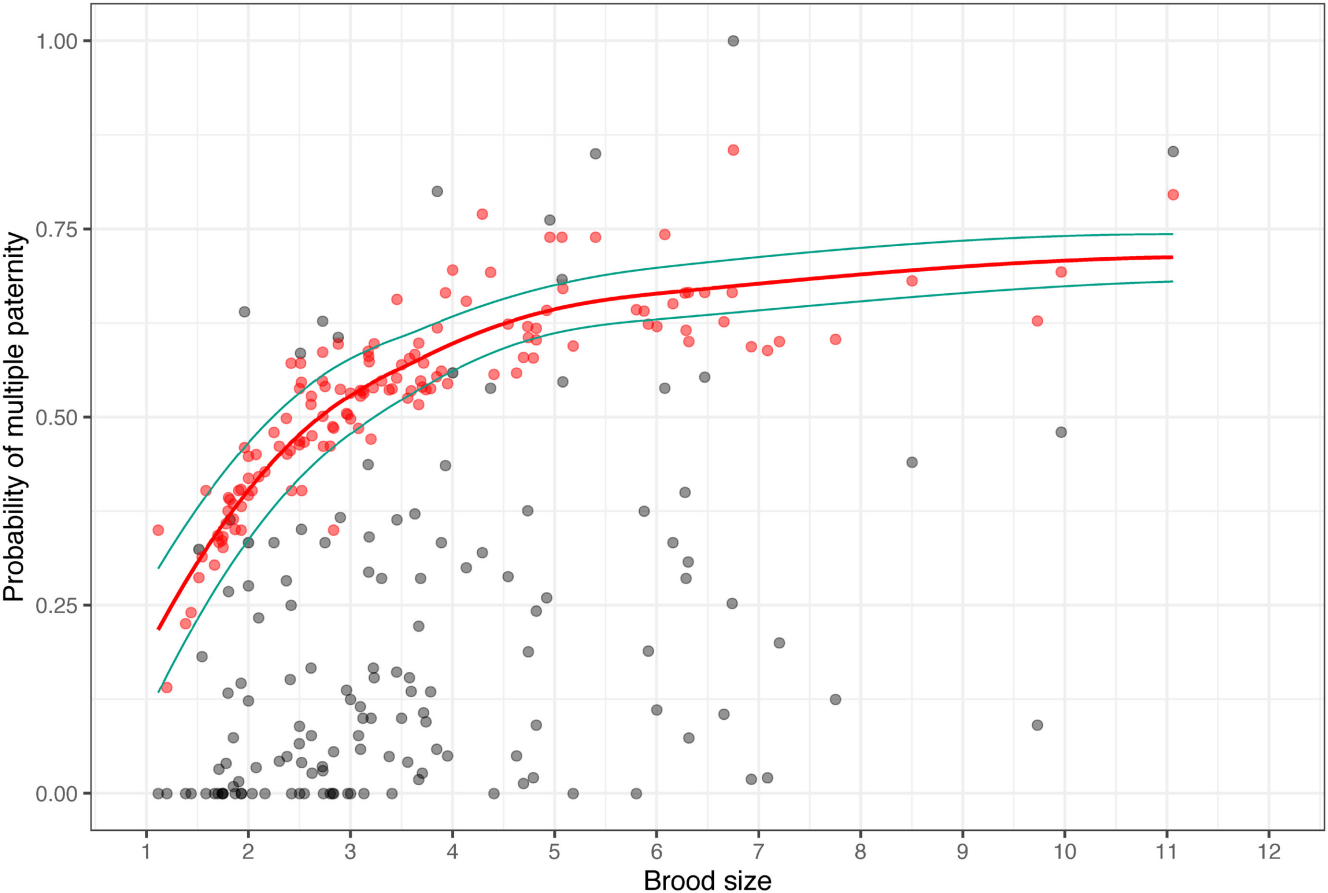
Null model ($p_{B}$) and field and laboratory estimates (p) of the probability of multiple paternity over different average brood sizes in bird populations, from studies that used microsatellite DNA results. Mean null model estimates are shown with a red line and 95% credible interval with green lines. Expected null model probabilities for individual populations are red dots. Dark gray dots are the field and laboratory estimates.

**FIGURE 3:**
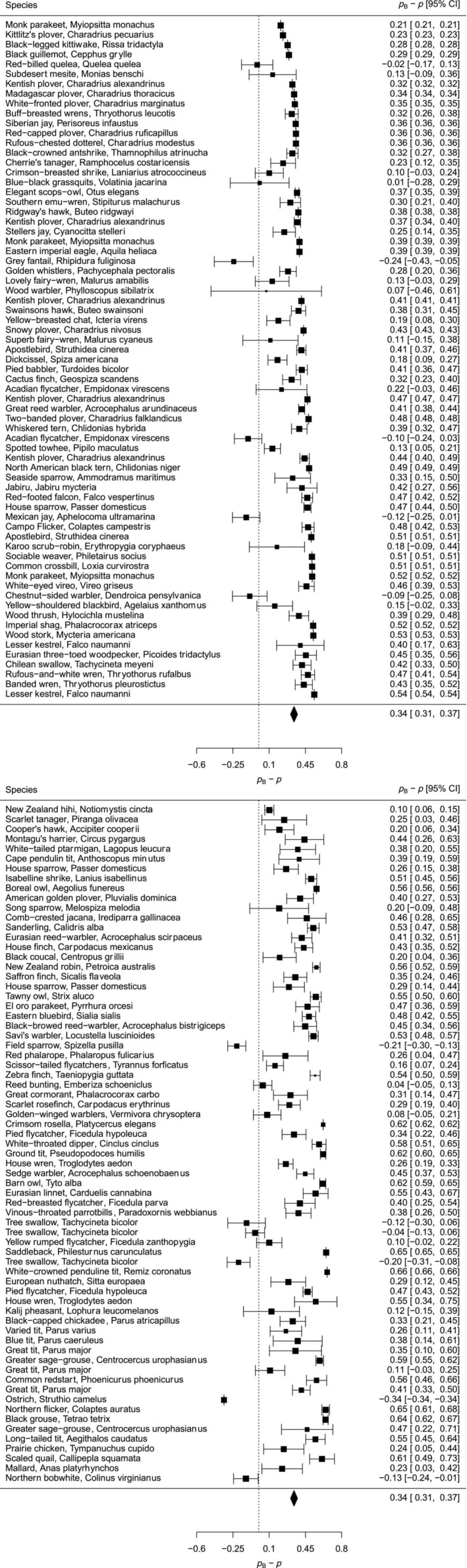
Forest plots of the deviation of probability of multiple paternity from the null models estimate ($p_{B}‐p$) for 138 bird populations using microsatellite DNA to identify multiple paternity. Populations are in order from smallest to largest mean brood size (range of 1.1 to 11.1 chicks/brood).

For the studies of Avian species that used microsatellite DNA, our null model exhibited a positive curvilinear predicted pattern of change in expected probability of multiple paternity ($p_{B}$) with mean brood size among populations of birds (Figure 2). The predicted multiple paternity under the null model varied between 0.14 and about 0.85. For 92% of the bird populations, the observed probability of multiple paternity fell below the predicted probabilities under our null model. Only 11 of 138 populations (8.0%) exhibited observed values of multiple paternity above the mean model predictions, the latter being conservative estimates of predicted probability of multiple paternity under the assumption of equal likelihood of siring success for mating males (Abebe et al., 2019; Correia et al., 2021; Dobson et al., 2018). For deviations in multiple paternity from the null model, credible intervals of 21 populations overlapped zero, indicating that at most, only 15.2% of the populations were well‐described by the null model (Figure 3). The mean deviation from the null model was 0.340, or 34.0‐percentage points below the null model expected probabilities of multiple paternity under equal fertilization success by mating males.

We compared 104 socially monogamous bird populations to 31 populations that exhibited other types of behavioral mating systems (viz., polygynous, polyandrous, polygynandrous, and “variable”; three populations were not behaviorally defined). The odds of single paternity (viz., *p* = 0) did not significantly differ between socially monogamous/paired species and other types of behavioral mating systems (CMH test, COR = 0.85, *p* = .78). We tested whether behaviorally monogamous/paired populations had different probabilities of multiple paternities than populations with nonmonogamous mating systems, and the slight difference was opposite to the expected direction and not significant (means = 20.7% monogamous and 19.8% nonmonogamous; clustered Wilcoxon test, *W* = 0.83, *p* = .07). Likewise, the mean deviations of null model predictions and real probabilities of multiple paternity were trivial and not significant (32.1 and 32.8‐percentage points, respectively; clustered Wilcoxon test, *W* = 0.76, *p* = .80). We used the combined sample of 138 populations in further analyses.

### 3.2 Comparison of birds and mammals

Among the bird species that used microsatellite DNA (*n* = 120 species), a few species had studies of more than one population (11/120 = 9.2% of the species, a mean of 2.64 populations per species, range = 2 to 5 populations). The probability of multiple paternity was moderately consistent among species with more than one population studied (*α* = 0.40; 95% CI [0.01, 0.69]), while mean brood size was substantially consistent (*α* = 0.83; 95% CI [0.62, 0.94]). Both variables for the three mammalian species that were each measured twice were not significantly consistent for probability of multiple paternity (*α* = 0.65; 95% CI [−0.67, 0.75]) and for mean brood size (*α* = 0.88; 95% CI [−0.67, 0.95]). Because of the relatively low agreement between the observed probabilities of multiple paternity among populations of the same species for both birds and mammals, we compared bird and mammal results using populations as the sampling unit.

**FIGURE 4:**
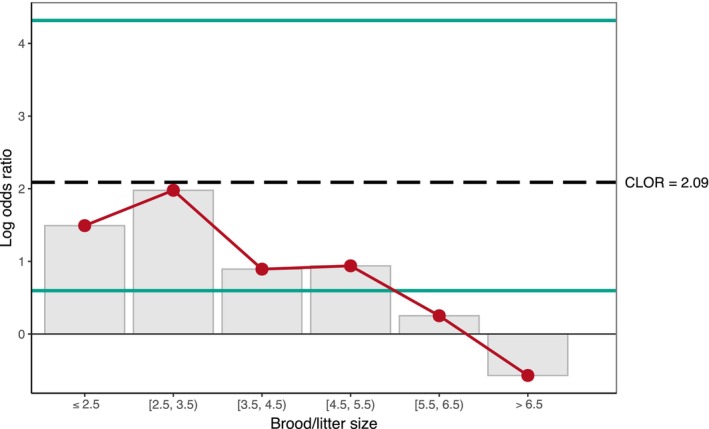
Point estimates for conditional log odds ratios (LOR) of zero multiple paternity in birds versus mammals, when controlling for brood/litter size in clusters. Common log odds ratios (CLOR) across brood/litter size clusters (dashed black lines) and 95% confidence intervals (solid green lines) are indicated in each.

**FIGURE 5:**
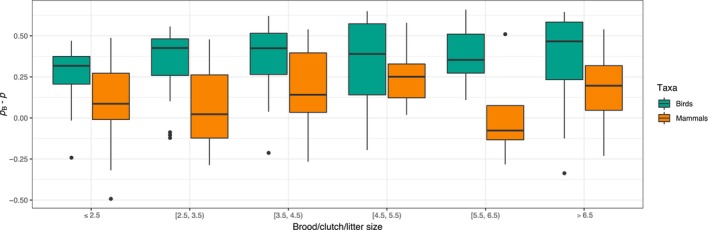
Comparison of birds (green, 138 populations using microsatellite DNA) and mammals (orange, 64 populations using microsatellite DNA) for the deviation of field and laboratory estimates of probability of multiple paternity from the null model predictions at different brood and litter sizes clustered by integer intervals. Bars indicate the mean deviation of the observed values of multiple paternity from the null model predictions ($p_{B}-p$). Boxes represent the interquartile range (IQR), and upper and lower whiskers extend to the largest and smallest values within 1.5 × IQR from the 75th and 25th percentiles, respectively. Black points indicate outliers.

Observed probabilities of multiple paternity in birds were smaller than probabilities of multiple paternity observed in mammals across clustered brood/litter sizes (respectively, means = 19.5% and 45.6%, *n* = 138 and 64; clustered Wilcoxon test, *W* = 1.73, *p* < .01). Two of 64 (3.1%) mammalian populations and 29 of 138 (21.0%) bird populations had an estimated probability of multiple paternity of zero. Thus, avian populations analyzed with the microsatellite DNA technique were on average more than eight times as likely to give a zero probability of multiple paternity compared to mammalian populations across brood/litter size clusters (CMH test, COR = 8.07, *p* < .01). Conditional LOR ranged from – −0.57 to 1.98 when controlling for brood/litter size clusters (Figure 4). Deviations from the null model, in percentage points, were more than twice as large for birds than for mammals (respectively, mean of 34.0 and 13.6‐percentage points), and deviations from the null model for birds were significantly larger than those of mammals when accounting for clustered brood/litter sizes (Figure 5; clustered Wilcoxon test, *W* = 2.20, *p* < .01). Finally, mean numbers of sires for bird populations were significantly smaller than those for mammals, at 1.25 for birds and 1.60 for mammals (*t* = −4.97, *p* < .01). Mean brood and litter sizes, however, were similar (3.64 brood size and 3.92 litter size, *t* = −1.06, *p* = .29). Numbers of broods and litters sampled did not differ significantly, though samples of broods averaged about a third higher (means = 51.2 broods and 37.7 litters per population, *t* = 1.86, *p* = .06).

### 3.3 Phylogenetic contrasts

Phylogenetic mixed models with no fixed effects covariates revealed a small‐to‐medium association between multiple paternity and the phylogeny for birds (*h* = 0.27, 95% CI [0.09, 0.50]) and mammals (*h* = 0.28, 95% CI [0.08, 0.56]). A medium association was evident for deviations from the null model for birds (*h* = 0.41, 95% CI [0.06, 0.72]) and mammals (*h* = 0.58, 95% CI [0.15, 0.89]). Historical patterns of multiple paternity across the combined phylogenetic tree were more apparent than in the separate phylogenies (Figure 1; *h* = 0.39, 95% CI [0.21, 0.58]), and this was also the case for the deviations from the null model (*h* = 0.62, 95% CI [0.34, 0.82]). For comparison and as expected, body mass for birds and mammals exhibited strong associations with the phylogenies in our samples (*h* = 0.82, 95% CI [0.72, 0.88]) and (*h* = 0.93, 95% CI [0.86, 0.97], respectively).

Within birds, the phylogenetic‐adjusted mating comparison indicated that the odds of multiple paternity in nonmonogamous species were not significantly different from the odds of multiple paternity in monogamous species (LOR = 0.29, 95% CI [−0.51, 1.07]). The mean phylogeny‐adjusted difference from null model predictions between nonmonogamous species and monogamous species was also not significantly different (*δ* = −0.04, 95% CI [−0.14, 0.06]).

When comparing birds and mammals with an adjustment for phylogeny, the likelihood of exhibiting multiple paternity was not significantly different between mammalian and bird species (LOR = 1.95, 95% CI [−1.72, 5.56]). The phylogeny‐adjusted deviations from null model predictions of multiple paternity in mammal species were not significantly different from those of bird species (*δ* = −0.17, 95% CI [−0.78, 0.45]).

## 4 DISCUSSION

Our null model, based on an equal chance of success at siring offspring among a female's mates, gives a reasonable expectation of the pattern of multiple paternity across litter sizes in the absence of biological influences that would result in unequal siring success among mates for comparison to the real results of multiple paternity and brood size among observed populations (Abebe et al., 2019; Correia et al., 2021; Dobson et al., 2018). Thus, the purpose of the null model was not to describe or “fit” the observed data. Rather, the null model was used to estimate the values of multiple paternity that would be seen in a mating system where environmental and evolutionary constraints that likely restrict rates of multiple paternity do not exist. Under the lack of these constraints, we can reasonably expect variation in sires for populations where the average number of sires approaches one or approaches the generated brood or litter size to be greater than the observed variation in sires (which approaches zero in available data for some of these populations).

For birds, the null model gives a curvilinear relationship between the probability of multiple paternity and mean brood size, which falls between about 25% and 70% multiple paternity, depending on the mean brood size (Figure 2). The null model predicts that multiple paternity will increase with increases in brood size at relatively low values of the latter and should level off at brood sizes greater than about 10 offspring. Deviations from the null model average about 34.0‐percentage points (Figure 3), with only about 15% of avian populations falling close to predictions of the null model under equal fertilization success among mating males. Dobson et al. (2018) found very similar null model predictions for mammalian species at similar litter sizes, although deviations from the null model were less, at 29.0‐percentage point deviations from the null model, and with 30% of populations falling close to the null model predictions. The leveling off of the expected probability of multiple paternity at higher brood sizes of about 10 was common to birds and mammals and was confirmed at somewhat higher values for reptiles, fish, and invertebrates by Correia et al. (2021).

Results from avian studies revealed probabilities of multiple paternity were extremely low compared to predicted probabilities of multiple paternity under the null model, with an average of about 20% of broods in microsatellite DNA analyses exhibiting multiple paternity. As well, significantly more analyses of birds found no multiple paternity when compared to mammals. While review studies have examined possible causes of the “frequent occurrence” of extra‐pair paternity in birds (Brouwer & Griffith, 2019; Griffith et al., 2002; Jennions & Petrie, 2000; Westneat & Stewart, 2003), a more general question might be why bird populations exhibit such low probabilities of multiple paternity. We found the probability of multiple paternity to be low in comparison to predictions under the null model's assumption of equal chance of success among a female's mates, compared to the much higher probabilities among mammals, and compared to reports from other animal taxa (Correia et al., 2021).

One hypothesis is that parental care by both sexes is required for successful reproduction and that associated pair bonding between the sexes might explain the low probability of multiple paternity in socially monogamous bird species (Ball et al., 2017; Birkhead & Møller, 1996; Griffith et al., 2002; Maher et al., 2017). The proportion of bird species identified as socially monogamous has been estimated at 90% (Lack, 1968) and 81% (Cockburn, 2006). For birds, however, we found little difference in the probability of multiple paternity between social monogamous populations and those exhibiting other types of mating systems. Further, the probability of multiple paternity can be quite high in pair‐bonded species (e.g., 26% of young were not sired by the strongly bonded male in California towhees, *Pipilo crissalis*; Benedict, 2014). Among mammals, social monogamy is relatively rare, at about 9% of the species (Lukas & Clutton‐Brock, 2013). No evidence of paternal care was found in the closely associated pairs of many of the socially monogamous mammalian species (about 41% of 229 species).

A few processes might lead to low probabilities of multiple paternity in populations. One is sexual selection (e.g., male–male competition) that restricts the number of mates for females and thus reduces opportunities for multiple paternity (Correia et al., 2021). A second possible influence is postmating factors, such as the order of male mates with respect to the timing of female receptivity to fertilization, differences in the amount of sperm deposited by males, and the associated number of copulations. For birds, sexual selection has long been documented, and it has been demonstrated or assumed to produce sexual dichromatism and other sexual dimorphisms in ornaments (Andersson, 1994). In addition, social monogamy has been assumed to limit the number of mating partners for bird species, despite their high mobility (Emlen & Oring, 1977). Other factors that might limit access to mates in birds might include environmental constraints, such as low density and small home ranges, though support for this hypothesis among mammalian species is weak (Dobson et al., 2010).

A comparison of a pair of well‐studied species provides anecdotal support for the hypothesis that the species with the greatest deviations from the null model exhibited the strongest sexual selection (Correia et al., 2021). This hypothesis suggests that the species with the lowest probabilities of multiple paternity should exhibit the highest degrees of sexual dimorphism, and vice versa. Tree swallows (*Tachycineta bicolor*) exhibited a somewhat greater degree of multiple paternity than expected from the null model (Figure 3). They nest in dispersed cavities, and the sexes are very similar in appearance (Robertson et al., 1992). By contrast, barn swallows (*Hirundo rustica*) deviate moderately from the null model (deviations of −21.9 and −25.0‐percentage points in two studies using DNA fingerprinting; Møller & Tegelström, 1997; Smith et al., 1991). They exhibit both ornaments (long tail feathers) and size dimorphism that favors males, suggesting strong sexual selection. Several studies of sexual selection on males have born this out (reviewed by Møller, 1988, 1994). Unfortunately, few examples exist of closely related bird species where both sexual selection and multiple paternity have been well studied.

Social monogamy in avian species may have a strong influence on the probability of multiple paternity. However, conditions that influence male defense of females during the period of sexual receptivity per se will likely prove most limiting on multiple paternity. A good example might be results on mate defense when females are most receptive, behaviorally and physiologically, to fertilization. In mammals, sexual size dimorphism appears to give a reasonable indication of the most sexually selected species, and those are the species that are most limited in the probability of multiple paternity (Correia et al., 2021). In the New Zealand tui (*Prosthemadera novaeseelandiae*), male body size was strongly associated with male success within the pair‐bond, such that EPPs declined significantly as male size increased (Wells et al., 2015). Sexual dimorphisms, however, may not be the best indicator of the intensity of sexual selection in birds because there are many causes of observable differences between males and females (Owens & Hartley, 1998; Selander, 1972). Thus, the combination of behavioral studies of sexual selection and multiple paternity may provide the best tests of the thesis that strong sexual selection produces low probabilities of multiple paternity in populations (Roeder et al., 2019).

Our results matched those found in numerous other taxa in which probabilities of multiple paternity are considerably less than expected under mating with an equal chance of siring success (Correia et al., 2021). This is especially so for mammals, which exhibited similar ranges of litter sizes as brood sizes of birds, and where a sufficient sample of populations has been studied to allow quantitative comparison. Across clusters of brood and litter sizes, birds deviated from the null model much more than mammals. Reptiles deviated from the null model almost twice as much as mammals, while fish and invertebrate species deviated by much less, about half the deviation found in mammals (Correia et al., 2021).

Within birds, the phylogenetic analyses produced results similar to our nonphylogenetic comparisons, thus supporting our conclusions. With respect to the influence of history on multiple paternity and on its deviations from our null model, we had no clear a priori expectation or gauge for what would constitute a strong association with phylogeny. Thus, we examined the historical pattern of body mass, which has a strong association with the pattern of phylogeny in both birds and mammals (respectively, Dobson et al., 2021; Dobson & Oli, 2007). Multiple paternity and deviations from our null model did not appear to be strongly influenced by the historical patterns of the phylogenies when compared to the highly phylogenetically conserved body mass trait of our sampled species. However, even the weak to moderate influences of evolutionary history on patterns of multiple paternity and deviations of multiple paternity from our null model make analyses using phylogenetic adjustments seem reasonable.

Despite the expectation that more flexible mating systems than social monogamy would have greater opportunities for multiple paternity in birds, thus deviating less from our null model, this was not true for the bird species represented in our data. When bird and mammal species were compared, mammals had an 8‐fold greater chance of exhibiting multiple paternity compared to birds, which was significant. This difference, however, was not significant when adjusted for phylogeny. Additionaly, bird species showed a greater deviation from the null model compared to mammalian species, but this difference was also no longer significant when adjusted for the phylogeny. This lack of significant differences between the classes should not be surprising, because a fundamental difference between these groups is their divergent evolutionary histories, and thus differences are part of the phylogeny, which is treated as a random variable in the phylogenetic analyses. At the same time, the heritability of multiple paternity and the deviations from the null model increased substantially for the combined tree over the heritability of these values when examined for the separate Mammalia and Aves trees. This result is expected when the two major branches of the combined tree differ in trait values, thus revealing similar patterns of differences between birds and mammals as in the nonphylogenetic analyses.

In the present study, associations of multiple paternity with such life‐history traits as juvenile or adult mortality rates were not considered (Arnold & Owens, 2002; Beck et al., 2020; Sakao et al., 2019; Wells et al., 2015; Wink & Dyrcz, 1999). Important ecological variables such as home range size, population density, and habitat type might also be associated with the degree of multiple paternity (Biagolini‐Jr et al., 2017; Dobson et al., 2010; Griffith et al., 2002). Finally, we did not study such possible biases in data collection as latitude or geographic location, or search for biogeographic patterns (Bonier et al., 2014; Valcu et al., 2021). These topics require future investigation, as they have already proved enlightening in studies of extra‐pair paternity (reviewed by Brouwer & Griffith, 2019). While samples of species that had observations for multiple populations were small, populations did not show strong consistency in multiple paternity within species. Additionally, the associations of the probability of multiple paternity with both the bird and mammalian phylogenies were relatively weak. These results suggest that multiple paternity may be highly variable and perhaps respond flexibly to social and ecological circumstances in local environments.

## AUTHOR CONTRIBUTIONS

**F. Stephen Dobson:** Conceptualization (equal); data curation (lead); methodology (equal); writing – original draft (equal); writing – review and editing (equal). **Hannah E. Correia:** Conceptualization (equal); formal analysis (equal); methodology (equal); writing – original draft (equal); writing – review and editing (equal). **Ash Abebe:** Conceptualization (equal); formal analysis (equal); writing – original draft (equal); writing – review and editing (equal).

## Supporting information


Appendix 1.

Appendix A.

Appendix B.

## Data Availability

Code and data to replicate the analyses described in this paper have been made available on the Harvard Dataverse repository at: https://doi.org/10.7910/DVN/IRAKE9.
