# Supplementary material for: How much multiple paternity should we expect? A study of birds and contrast with mammals
Source: Ecol Evol. 2024 Mar 1;14(3):e11054. doi: 10.1002/ece3.11054 (PMC10905237; doi:10.1002/ece3.11054)
Supplement: Supplementary file 1 — Appendix 1. [file ECE3-14-e11054-s001.docx]

**Appendix 1**

**Section S1. Null model of multiple paternity**

Dobson et al. (2018) developed a combinatorial expression for calcuating the probability of multiple paternity in a litter under the assumption that all males who mated with a female had equal probability of siring each of her offspring. This formulation was based only on the number of offspring the female had in a litter and the female’s number of mates, the latter of which is a rarely observed or reported quantity in multiple paternity studies. Typically, only the number of sires identified within broods or litters and a single multiple paternity value representing the proportion of broods or litters with multiple paternity is reported. Therefore, Dobson et al. (2018) developed an alternative formulation of multiple paternity based on the probability of more than one sire occurring in a brood and only depending on the litter or brood size, number of sires, and a probability of paternity success that is equal for all sires (fig. S1). Our null model used observed mean number of sires and mean sizes of litters for each species to estimate the expected probability of siring success for each mating male, and then calculate a predicted probability of multiple paternity assuming the chance of siring success was equal among potential sires.

Figure S1. Null model and data analysis procedure from Dobson et al. 2018; Abebe et al. 2019; Correia et al. 2021 as applied to the meta-analytic comparison of multiple paternity in birds and mammals.


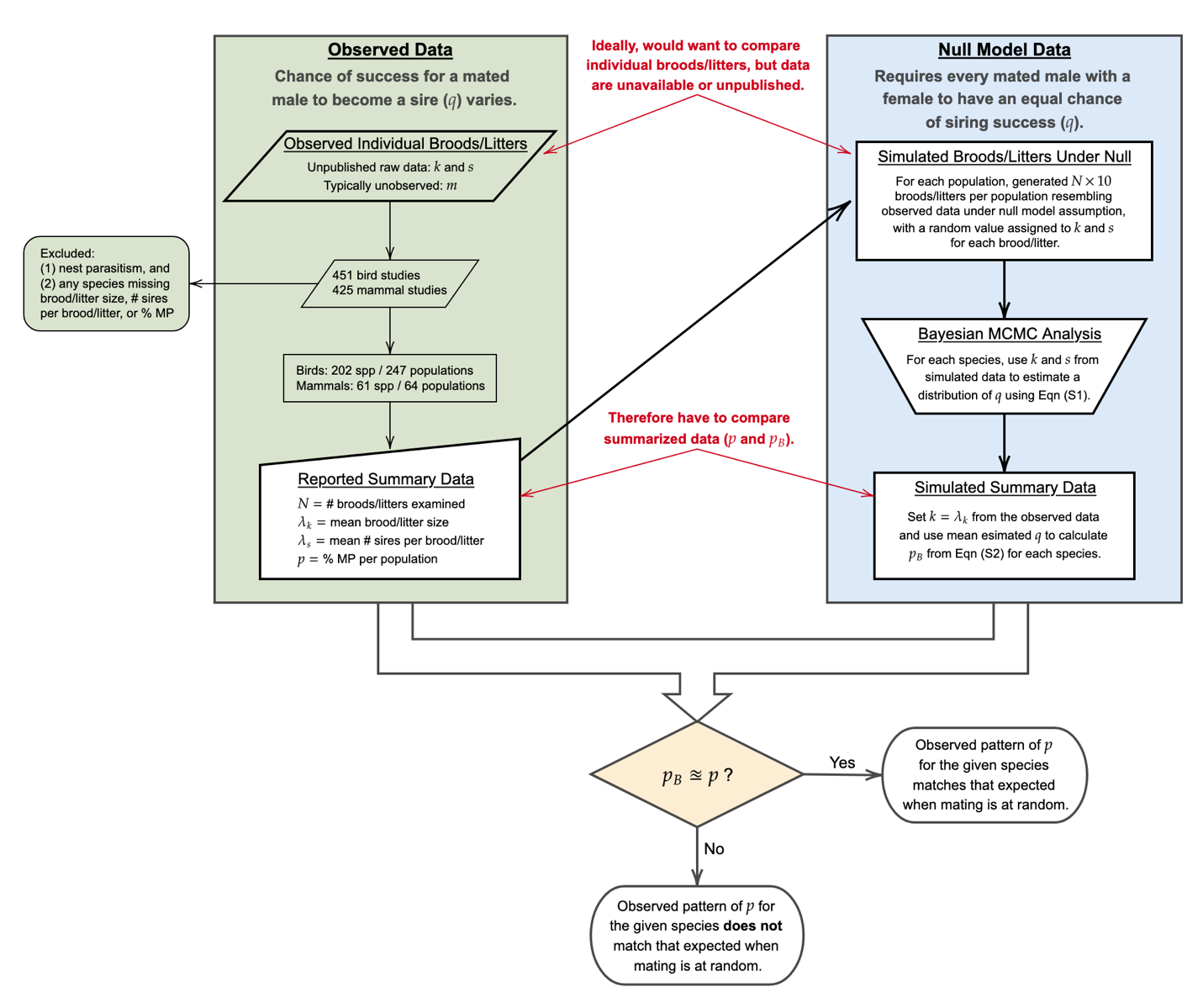


For each bird or mammal species, we used the mean brood or litter size and mean number of sires from the literature to generate brood or litter sizes and number of sire values under the null for a sample that was 10 times the population’s observed sample size (fig. S1; after Correia et al. 2021). The number of offspring in a brood or litter ($\boldsymbol{K}=\boldsymbol{k}$) cannot be less than one, and thus can be generated from a zero-truncated Poisson distribution with a rate parameter equal to the observed average brood or litter size ($\boldsymbol{\lambda}_{\boldsymbol{k}}$) (Plackett 1953). For each generated brood or litter, the number of sires (***S***) cannot be less than one, nor can it exceed ***k*** (***S = s, s = 1, 2, 3, …, k***), and can similarly be generated from a Poisson distribution with rate parameter equal to the observed average number of sires ($\boldsymbol{\lambda}_{\boldsymbol{s}}$) and truncated at zero and ***k*** (Finney 1947). Given ***k*** and ***q***, the probability of $\boldsymbol{S=s}$ follows a zero-truncated binomial distribution with probability mass function

| $P\left( S=s \vert k,q \right)=\frac{\binom{k}{s}q^{s}\left( 1-q \right)^{k-s}}{1-\left( 1-q \right)^{k}} ,$ | (S1) |
| --- | --- |

where $\boldsymbol{s=1,2,\ldots, k}$. As there is no closed-form solution for ***q*** in Equation S1, we estimated it numerically using a Bayesian model with Markov chain Monte Carlo (MCMC) sampling and a beta distribution (hyperparameters $\alpha=1$, $\beta=1$) as an uninformative prior for ***q***. The posterior estimate of ***q*** was derived from 10,000 simulations with two chains and 1000 iterations for the burn-in period. In cases where a species had more than one population studied, we assumed that distinct populations of the same species shared the same mechanisms generating patterns of multiple paternity for that species. Thus, we generated broods from all populations of a species combined into a single “superpopulation” and a single posterior distribution for ***q*** was estimated numerically for each species; mean ***q*** was calculated from this distribution.

With a mean ***q*** for each species, the expected probability of multiple paternity ($\boldsymbol{p}_{\boldsymbol{B}}$) is the probability that the number of sires is greater than 1, i.e., $\boldsymbol{P}\left( \boldsymbol{S}>1 \right)\boldsymbol{=1-P}\left( \boldsymbol{S=1} \right)$, thus

| $p_{B}=1-\frac{kq\left( 1-q \right)^{k-1}}{1-\left( 1-q \right)^{k}} ,$ | (S2) |
| --- | --- |

and can be calculated for each species by setting $\boldsymbol{k=}\boldsymbol{\lambda}_{\boldsymbol{k}}$ (fig. S1). This expression for $\boldsymbol{p}_{\boldsymbol{B}}$ for a given species is the probability of multiple paternity expected under mating at random with equal chance of siring success among mating males simulated by the random samples from the specified distributions of ***K*** and ***S*** used to estimate ***q***, and given the mean value of brood or litter size. For each population with frequency of observed multiple paternity ***p*** in nature, we calculated $\boldsymbol{p}_{\boldsymbol{B}}\boldsymbol{-p}$, which quantifies the deviation from the prediction of the null model.

In extreme cases where the observed mean number of sires approaches one or approaches the brood or litter size generated under the null model assumptions, the data generation procedure prior to the fitting of the null model produced values for the number of sires that are marginally higher than suggested by the reported mean number of sires in the literature. The truncated Poisson distribution (Finney 1946) used to generate values for the number of sires per brood from observational summary statistics produces data with an *untruncated* mean that is equal to the observed mean sires. The mean of a random sample drawn from the *truncated* distribution of the number of sires is therefore not equal, but rather is close to the observed mean number of sires from the real data (Plackett 1953). Data generated from the truncated Poisson distribution may thus introduce variation into the generated number of sires that was not observed in the field and laboratory data for populations, particularly with observed mean number of sires that were either close to one or close to the generated brood or litter size. Such was often the case in the populations of birds in our study, since many had observed mean number of sires that were close to one.

This feature of the null model for multiple paternity, however, was not an overlooked gap in the construction of the null model, and the null model data generation and estimation of $\boldsymbol{p}_{\boldsymbol{B}}$ is robust to extreme values of the number of sires (viz, when mean number of sires was near 1 or near the brood or litter size). Any statistical null model must start with some assumptions, and generating data from a truncated Poisson distribution for the number of sires is a biologically reasonable assumption for the null model system (Dobson et al. 2018; Abebe et al. 2019).

**Section S2. Comparison of DNA fingerprinting and microsatellite DNA in birds**

DNA fingerprinting generally produced fewer loci or alleles for identification of sires than microsatellite DNA analyses (Chambers et al. 2014), potentially making it easier to misidentify cases of multiple paternity as singly sired. Cases of zero probability of multiple paternity might therefore originate from three sources: from low detectability due to the laboratory technique applied, from truly low probabilities of multiple paternity, and from sampling variation, particularly in small populations. The Cochran-Mantel-Haenszel (CMH) test (Agresti 2002) was applied to determine whether the two molecular methods differed in detecting the presence of multiple paternity while controlling for brood size in birds. Average brood size was rounded into integer clusters (e.g., broods with average number of offspring between 1.5 and 2.5 were gathered into a single cluster representing average brood sizes around 2, while those between 2.5 and 3.5 were put into another cluster representing average brood sizes near 3, and so forth for the entire range of brood sizes in birds and mammals). Sample sizes for mean brood sizes less than 1.5 (n = 5 analyses using fingerprinting, n = 4 analyses using microsatellites), and for mean brood sizes greater than 7.0 (n = 4 analyses using fingerprinting, n = 7 analyses using microsatellites) were low. Therefore, mean brood sizes of at most 2.5 were grouped into a single cluster, and mean brood sizes greater than 6.5 were also grouped into a single cluster. A 2 × 2 × 6 contingency table was thus constructed with the counts of analyses that had zero and nonzero multiple paternity for each of the two methods across six clusters of brood sizes. The conditional log odds ratios (LOR) were calculated for each brood size cluster, and the common odds ratio (COR) and common log odds ratios (CLOR) were calculated overall, corresponding to the test of independence using the CMH procedure. Significance of the LOR and CLOR was calculated using an exact permutation test.

Studies of bird populations that used DNA fingerprint and microsatellite DNA techniques were also tested for a significant difference in the proportions of multiple paternity using a clustered Wilcoxon rank sum test (Datta and Satten 2005), where brood size was clustered as for the CMH test. An approximation of an exact permutation test using 2000 random permutations was used to calculate significance of the clustered Wilcoxon rank sum test.

DNA fingerprinting techniques were applied to 109 populations (number of broods per population ranging from 4 to 162), and 48 of these (44.0%) estimated a multiple paternity probability of zero. For populations with analyses of microsatellite DNA, only 29 of 138 (21.0%) estimated the probability of multiple paternity at zero (range in sample size of broods from 3 to 485). The log odds of the probability of multiple paternity being estimated at zero ranged from 0.67 to 2.00 when brood size cluster was controlled (fig. S2) and averaged about three times as high in populations with DNA fingerprinting-based analyses as they averaged in populations with microsatellite DNA analyses across brood size clusters (CMH test, COR = 2.95, P < 0.01). Across clustered brood sizes, the overall mean probability of multiple paternity was nearly twice as high for populations analyzed with microsatellite DNA techniques as for those analyzed with DNA fingerprinting techniques (respectively, means = 19.5% and 10.8%, n = 138 and 109, clustered Wilcoxon test, W = 1.99, P < 0.01).

Further differences were apparent between other multiple paternity-related variables. Estimated number of sires was greater for populations analyzed using microsatellite DNA than for those using DNA fingerprinting (respectively, means = 1.25 and 1.12, n = 138 and 109, t = 3.82, P < 0.01). Sample sizes in populations analyzed using microsatellite DNA averaged about 73% larger (respectively, 51.2 and 29.6, n = 138 and 109, t = 3.95, P < 0.01). However, mean brood sizes in populations with microsatellite DNA analyses were similar to those with DNA fingerprinting analyses (respectively, means = 3.6 and 3.4, n = 138 and 109, t = 1.24, P = 0.22).

Finally, deviations in multiple paternity from our null model predications were similar for populations with microsatellite DNA analyses and those with DNA fingerprinting analyses when accounting for clustered brood/litter sizes (respectively, 33.1 and 38.9-percentage points below model predictions, n = 138 and 109, clustered Wilcoxon test, W = 1.72, P = 0.70). Since populations with DNA fingerprinting analyses were significantly more likely to yield probabilities of multiple paternity of zero, microsatellite DNA is the preferred technique for measuring paternity (Chambers et al. 2014), and studies of both birds and mammals reported microsatellite DNA results, we limited further analyses to studies using microsatellite DNA techniques.

Figure S2. Point estimates for conditional log odds ratios (LOR) of zero multiple paternity in DNA fingerprinting versus microsatellite DNA studies, when controlling for brood/litter size in clusters. Common log odds ratios (CLOR) across brood/litter size clusters (dashed black lines) and 95% confidence intervals (solid green lines) are indicated in each.

**Section S3. Phylogenetic mixed models**

We downloaded 10,000 phylogenetic trees from VertLife for both birds and mammals (<http://vertlife.org/phylosubsets/>) and generated a consensus tree for each using the coalescent method (Jetz et al. 2012; Upham et al. 2019). A phylogenetic tree for Amniota was obtained from TimeTree (<http://www.timetree.org>; Kumar et al. 2022), onto which the bird and mammal phylogenies were grafted to create a single combined tree for both birds and mammals (fig. 1). Associations of multiple paternity and deviations from the null model with the phylogenetic patterns were estimated with Bayesian phylogenetic models using the *brms* package in R (Bürkner 2017; R Core Team 2022). The phylogenetic model

| $g(E(Y_{A}\vert\boldsymbol{a}_{A}))=\beta_{A}+\boldsymbol{a}_{A}\boldsymbol{+}\boldsymbol{\varphi}_{A[\text{pop}]}$ | (S3) |
| --- | --- |

was fit for birds, where $\beta_{A}$ is the intercept, $\boldsymbol{a}_{A}$ represents the variation from the additive genetic effects calculated from the phylogenetic tree of avian species, $\boldsymbol{\varphi}_{A[\text{pop}]}\boldsymbol{\sim}N(\boldsymbol{0},\tau_{A}^{2}\mathbf{I}_{A})$ is a random effect that accounts for differences among populations of the same species, and $g\left( \cdot\right)$ is a link function which is taken as the logit function for binary responses and identity function for Gaussian responses. The response distribution is taken to be $Y_{A}\sim B(n_{A},p_{A})$ for the number of instances of multiple paternity in $n_{A}$ broods with probability $p_{A}$ of multiple paternity for an avian species and $Y_{A}\sim N(\mu_{A},\sigma_{A}^{2})$ for deviations in avian multiple paternity probabilities from those of the null model. Next, the model

| $g\left( E\left( Y_{M} \vert\boldsymbol{a}_{M} \right) \right)=\beta_{M}+\boldsymbol{a}_{M}\boldsymbol{+}\boldsymbol{\varphi}_{M[\text{pop}]}$ | (S4) |
| --- | --- |

was fit for mammalian species, with intercept $\beta_{M}$, variation from the additive genetic effects calculated from the phylogenetic tree of mammalian species $\boldsymbol{a}_{M}$, random effect $\boldsymbol{\varphi}_{M}\boldsymbol{\sim}N(\boldsymbol{0},\tau_{M}^{2}\mathbf{I}_{M})$ that accounts for multiple populations within a species. Similar to the avian case, we took $Y_{M}\sim B(n_{M},p_{M})$ when studying the number of instances of multiple paternity, where $p_{M}$ is the probability of multiple paternity for a mammalian species in $n_{M}$ litters, and $Y_{M}\sim N(\mu_{M},\sigma_{M}^{2})$ when the response of interest was deviation in probability of multiple paternity from that of the null model. For the combined data including both taxa, we fit the model

| $g\left( E\left( Y_{C} \vert\boldsymbol{a}_{C} \right) \right)=\beta_{C}+\boldsymbol{a}_{C}\boldsymbol{+}\boldsymbol{\varphi}_{C[\text{pop}]}$ | (S5) |
| --- | --- |

where $\beta_{C}$ is the intercept, $\boldsymbol{a}_{C}$ represents the variation from the additive genetic effects calculated from the combined phylogenetic tree of bird and mammal species, $\boldsymbol{\varphi}_{C}\boldsymbol{\sim}N(\boldsymbol{0},\tau_{C}^{2}\mathbf{I}_{C})$ is the random effect accounting for differences among populations of the same species. Again $Y_{C}\sim B(n_{C},p_{C})$ for number of instances of multiple paternity where $p_{C}$ is the probability of multiple paternity for a given species in $n_{C}$ litters or broods and $Y_{C}\sim N(\mu_{C},\sigma_{C}^{2})$ for deviations in multiple paternity from the null model. The additive genetic effects are assumed to be normally distributed; so, $\boldsymbol{a}_{A}\boldsymbol{\sim}N(\boldsymbol{0},\omega_{A}^{2}\boldsymbol{\Omega}_{A})$, where $\boldsymbol{\Omega}_{A}$ is the phylogenetic correlation matrix for avian species, and $\boldsymbol{a}_{M}\boldsymbol{\sim}N(\boldsymbol{0},\omega_{M}^{2}\boldsymbol{\Omega}_{M})$, where $\boldsymbol{\Omega}_{M}$ is the phylogenetic correlation matrix for mammalian species. Similarly, $\boldsymbol{a}_{C}\boldsymbol{\sim}N(\boldsymbol{0},\omega_{C}^{2}\boldsymbol{\Omega}_{C})$, where the phylogenetic correlation matrix for all species (both avian and mammalian) is represented by $\boldsymbol{\Omega}_{C}$.

We also conducted a phylogenetic comparative analysis of mean body mass for the bird and mammal species. The Bayesian phylogenetic models in Equations (S3) and (S4) were fit for body mass of birds and mammals, respectively, where $Y_{M}\sim Exp\left( \lambda_{M} \right)$ and $Y_{A}\sim Exp\left( \lambda_{A} \right)$ and $g\left( \cdot\right)$ is a log link function.

We calculated the heritability of the probability of multiple paternity, the deviations of multiple paternity from the null model, and body mass across species within each taxon as

$$h=\frac{\omega^{2}}{\omega^{2}+\tau^{2}+\nu}$$

where $\omega^{2}$ is the additive genetic variance, $\tau^{2}$ is the random effects variance, and $\nu$ is the distribution-specific variance (Ives and Helmus, 2011). For models where the response is Gaussian, $\nu=\sigma^{2}$; when the response is modeled as a binomially distributed variable with logit link, $\nu=\pi^{2}/3$; and when the response is modeled as an exponentially distributed variable with log link, $\nu=1$ (Nakagawa et al. 2017).

We also examined differences in multiple paternity and deviations from the null model between socially monogamous and non-socially monogamous species using the following phylogenetic mixed model:

| $g(E(Y_{A}\vert\boldsymbol{a}_{A}))=\beta_{A}+\delta_{A}x_{A}+\theta_{A}{log}_{10}\left( b_{A} \right)+\boldsymbol{a}_{A} \boldsymbol{+}\boldsymbol{\varphi}_{A[\text{pop}]},$ | (S6) |
| --- | --- |

where $g(\boldsymbol{\cdot})$, $\beta_{A}$, $\boldsymbol{a}_{A}$ and $\boldsymbol{\varphi}_{A[\text{pop}]}$ are as in Equation (S3); $\delta_{A}$ and $\theta_{A}$ are coefficients; $x_{A}$ is an indicator function where $x_{A}=1$ for non-monogamous bird species and $x_{A}=0$ for monogamous bird species; and $b_{A}$ is the average avian brood size. To estimate the differences between birds and mammals in values of multiple paternity and deviations from the null model after accounting for the phylogenetic effects, we fit the model

| $g(E(Y_{C}\vert\boldsymbol{a}_{C}))=\beta_{C}+\delta_{C}x_{C}+\theta_{C}{log}_{10}\left( b_{C} \right)+\boldsymbol{a}_{C} \boldsymbol{+}\boldsymbol{\varphi}_{C[\text{pop}]},$ | (S7) |
| --- | --- |

where $g\left( \boldsymbol{\cdot} \right)$ is again a link function; $\beta_{C}$ and $\boldsymbol{a}_{C}$ and $\boldsymbol{\varphi}_{C[\text{pop}]}$ are as in Equation (S5); $\delta_{C}$ and $\theta_{C}$ are coefficients; $x_{C}=1$ to indicate mammalian species and $x_{C}=0$ for bird species; and $b_{C}$ is the average brood or litter size. The fixed terms $b_{A}$ and $b_{C}$ are to account for multiple paternity varying with the mean number of offspring across species (Dobson et al. 2018). All statistical analyses were conducted in R 4.2.2 (R Core Team 2022).

**Appendix References**

Bürkner, P.-C. 2017. *brms*: an R package for Bayesian multilevel models using Stan.  Journal of Statistical Software 80:1-28.

Finney, D. J. 1947. The truncated binomial distribution. Annals of Eugenics 14:319–328. (doi: 10.1111/j.1469-1809.1947.tb02410.x)

Ives, A. R., and M. R. Helmus. 2011. Generalized linear mixed models for phylogenetic analyses of community structure. Ecological Monographs 81(3), 511-525.

Jetz, W., Thomas, G. H., Joy, J. B., Hartmann, K., and A. O. Mooers. 2012. The global diversity of birds in space and time. Nature 491:444–448.

Kumar, S., M. Suleski, J. E. Craig, A. E. Kasprowicz, M. Sanderford, M. Li, G. Stecher, and S. B. Hedges. 2022. TimeTree5: An Expanded Resource for Species Divergence Times. Molecular Biology and Evolution 39(8): msac174.

Nakagawa S., P. C. D. Johnson, and H. Schielzeth. 2017. The coefficient of determination R2 and intra-class correlation coefficient from generalized linear mixed-effects models revisited and expanded. J. R. Soc. Interface 14:20170213. http://doi.org/10.1098/rsif.2017.0213

Upham, N. S., J. A. Esselstyn, and W. Jetz. 2019. Inferring the mammal tree: species-level sets of phylogenies for questions in ecology, evolution, and conservation. PLoS Biology 17:e3000494.
