# Supplementary material for: How much multiple paternity should we expect? A study of birds and contrast with mammals
Source: Ecol Evol. 2024 Mar 1;14(3):e11054. doi: 10.1002/ece3.11054 (PMC10905237; doi:10.1002/ece3.11054)
Supplement: Supplementary file 2 — Appendix A. [file ECE3-14-e11054-s003.docx]

**Appendix A – Data References for Birds**

| **Species** | **Reference** |
| --- | --- |
| Acadian flycatcher, Empidonax virescens | Evans et al., 2009; Woolfenden et al., 2005 |
| Acorn woodpecker, Melanerpes formicivorus | Dickinson et al. 1995 |
| Adelie penguin, Pygoscelis adeliae | Pilastro et al. 2001 |
| Alpine accentor, Prunella collaris | Hartley et al. 1995; Heer 1996 |
| American golden plover, Pluvialis dominica | Yezerinac et al. 2013 |
| Apostlebird, Struthidea cinerea | Warrington et al. 2013; Woxvold and Mulder 2008 |
| Arabian babbler, Turdoides squamiceps | Lundy, Parker, and Zahavi 1998 |
| Australian magpie-lark, Grallina cyanoleuca | Hall and Magrath 2000 |
| Banded wren, Thryothorus pleurostictus | Cramer et al. 2011 |
| Barn owl, Tyto alba | Henry et al. 2013 |
| Barn swallow, Hirundo rustica | Møller and Tegelström 1997; Smith et al. 1991 |
| Barnacle goose, Branta leucopsis | Choudhury et al., 1993; Larsson et al., 1995 |
| Bell miner, Manorina melanophrys | Conrad et al. 1998 |
| Black coucal, Centropus grillii | Muck et al. 2009 |
| Black grouse, Tetrao tetrix | Alatalo et al. 1996; Lebigre et al. 2007 |
| Black guillemot, Cepphus grylle | Anker-Nilssen, Tycho 2010 |
| Black vulture, Coragyps atratus | Decker et al. 1993 |
| Black-browed reed-warbler, Acrocephalus bistrigiceps | Hamao & Saito, 2005 |
| Black-capped chickadee, Parus atricapillus | Mennill et al., 2004 |
| Black-crowned antshrike, Thamnophilus atrinucha | Tarwater et al., 2013 |
| Black-legged kittiwake, Rissa tridactyla | Helfenstein et al., 2004 |
| Blue duck, Hymenolaimus malacorhynchos | Triggs et al., 1991 |
| Blue tit, Parus caeruleus | Johannessen et al., 2005; Krokene, 1998 |
| Blue-black grassquits, Volatinia jacarina | Carvalho et al., 2006 |
| Blue-headed vireo, Vireo solitarius | Morton et al., 1998 |
| Boreal owl, Aegolius funereus | Koopman et al., 2007 |
| Brown skua, Catharacta lonnbergi | Millar et al., 1994 |
| Brown thornbill, Acanthiza pusilla | Green et al., 2002 |
| Buff-breasted wrens, Thryothorus leucotis | Gill et al., 2005 |
| Bull-headed shrike, Lanius bucephalus | Yamagishi et al., 1992 |
| Burrowing parakeet, Cyanoliseus patagonus | Masello et al., 2002 |
| Bushtit, Psaltriparus minimus | Bruce et al., 1996 |
| Cactus finch, Geospiza scandens | Petren et al., 1999 |
| Campo Flicker, Colaptes campestris | Dias et al., 2013 |
| Canary, Serinus canaria | Voigt et al., 2003 |
| Cape pendulin tit, Anthoscopus minutus | Ball et al., 2017 |
| Capricorn silvereye, Zosterops lateralis | Robertson et al., 2001 |
| Carolina wren, Thryothorus ludovicianus | Haggerty et al., 2001 |
| Cherrie's tanager, Ramphocelus costaricensis | Krueger et al., 2008 |
| Chestnut-sided warbler, Dendroica pensylvanica | Byers et al., 2004 |
| Chilean swallow, Tachycineta meyeni | Ferretti et al., 2016 |
| Chinstrap penguin, Pygoscelis antarcticus | Moreno et al., 2000 |
| Comb-crested jacana, Irediparra gallinacea | Haig, 2003 |
| Common crossbill, Loxia curvirostra | Kleven et al., 2008 |
| Common gull, Larus canus | Bukacińska et al., 1998 |
| Common loon, Gavia immer | Piper et al., 1997 |
| Common redstart, Phoenicurus phoenicurus | Kleven et al., 2007 |
| Common sandpiper, Actitis hypoleucos | Blomqvist et al., 2002; Mee et al., 2004 |
| Common starling, Sturnus vulgaris | Pinxten et al., 1993; Smith & von Schantz, 1993 |
| Common swift, Apus apus | Martins & Vasconcelos, 2009 |
| Common tern, Sterna hirundo | González-Solı́s et al., 2001; Griggio et al., 2004 |
| Cooper's hawk, Accipiter cooperii | Rosenfield et al., 2015 |
| Corn bunting, Miliaria calandra | Hartley et al., 1993 |
| Crimsom rosella, Platycercus elegans | Eastwood et al., 2018 |
| Crimson-breasted shrike, Laniarius atrococcineus | van den Heuvel et al., 2014 |
| Dickcissel, Spiza americana | Sousa & Westneat, 2013 |
| Dunnock, Prunella modularis | Burke et al., 1989 |
| Dusky antbird, Cercomacra tyrannina | Fleischer et al., 1997 |
| Eastern bluebird, Sialia sialis | Meek et al., 1994; Stewart et al., 2010 |
| Eastern imperial eagle, Aquila heliaca | Rudnick et al., 2005 |
| Eastern screech-owl, Megascops asio | Lawless et al., 1997 |
| El oro parakeet, Pyrrhura orcesi | Klauke et al., 2013 |
| Elegant scops-owl, Otus elegans | Hsu et al., 2006 |
| Eleonoras falcon, Falco eleonorae | Swatschek et al., 1993 |
| Eurasian dotterel, Eudromias morinellus | Owens et al., 1995 |
| Eurasian jackdaw, Corvus monedula | Henderson et al., 2000; Liebers & Peter, 1998 |
| Eurasian kestrel, Falco tinnunculus | Korpimäki et al., 1996 |
| Eurasian linnet, Carduelis cannabina | Bønløkke-Pedersen et al., 2002 |
| Eurasian oystercatcher, Haematopus ostralegus | Heg et al., 1993 |
| Eurasian reed-warbler, Acrocephalus scirpaceus | Davies et al., 2003 |
| Eurasian three-toed woodpecker, Picoides tridactylus | Li et al., 2009; Pechacek et al., 2005 |
| Eurasian wryneck, Jynx torquilla | Wink et al., 2011 |
| European nuthatch, Sitta europaea | Segelbacher et al., 2005 |
| Field sparrow, Spizella pusilla | Celis-Murillo et al., 2017 |
| Fiordland penguin, Eudyptes pachyrhynchus | McLean et al., 2000 |
| Flammulated owl, Otus flammeolus | Arsenault et al., 2002 |
| Florida scrub-jay, Aphelocoma coerulescens | Quinn et al., 1999 |
| Galapagos hawk, Buteo galapagoensis | Faaborg et al., 1995 |
| Golden whistlers, Pachycephala pectoralis | van Dongen & Mulder, 2009 |
| Golden-winged warblers, Vermivora chrysoptera | Vallender et al., 2007 |
| Great cormorant, Phalacrocorax carbo | Minias et al., 2016 |
| Great reed warbler, Acrocephalus arundinaceus | Hansson et al., 2004; Hasselquist et al., 1996; Leisler et al., 2001 |
| Great spotted woodpecker, Dendrocopos major | Michalek & Winkler, 2001 |
| Great tit, Parus major | García-Navas et al., 2015; Johannessen et al., 2005; Krokene, 1998; Strohbach et al., 1998; van Oers et al., 2008; Winkel et al., 2001 |
| Greater sage-grouse, Centrocercus urophasianus | Bird et al., 2013; Semple et al., 2001 |
| Grey fantail, Rhipidura fuliginosa | Hoffman et al., 2010 |
| Ground tit, Pseudopodoces humilis | Johannessen et al., 2011 |
| House finch, Carpodacus mexicanus | Lindstedt et al., 2007 |
| House martin, Delichon urbicum | Riley et al., 1995 |
| House sparrow, Passer domesticus | Cordero et al., 1999; Griffith et al., 1999; Václav & Hoi, 2007; Veiga & Boto, 2000 |
| House wren, Troglodytes aedon | Brylawski & Whittingham, 2004; Cramer et al., 2013; Soukup & Thompson, 1997 |
| Humboldt penguin, Spheniscus humboldti | Schwartz et al., 1999 |
| Imperial shag, Phalacrocorax atriceps | Calderón et al., 2012 |
| Isabelline shrike, Lanius isabellinus | Bao et al., 2019 |
| Jabiru, Jabiru mycteria | Lopes et al., 2013 |
| Kalij pheasant, Lophura leucomelanos | Zeng et al., 2016 |
| Karoo scrub-robin, Erythropygia coryphaeus | Ribeiro et al., 2012 |
| Kentish plover, Charadrius alexandrinus | Blomqvist et al., 2002; Maher et al., 2017 |
| Kittlitz's plover, Charadrius pecuarius | Maher et al., 2017 |
| Laughing kookaburra, Dacelo novaeguineae | Legge & Cockburn, 2000 |
| Lesser gray shrike, Lanius minor | Valera et al., 2003 |
| Lesser kestrel, Falco naumanni | Alcaide et al., 2005; Negro et al., 1996 |
| Little owl, Athene noctua | Müller et al., 2001 |
| Long-eared owl, Asio otus | Marks et al., 1999 |
| Long-tailed tit, Aegithalos caudatus | Hatchwell et al., 2002 |
| Lovely fairy-wren, Malurus amabilis | Leitão et al., 2021 |
| Madagascar plover, Charadrius thoracicus | Maher et al., 2017 |
| Mallard, Anas platyrhynchos | Kreisinger et al., 2010 |
| Merlin, Falco columbarius | Warkentin et al., 1994 |
| Mexican jay, Aphelocoma ultramarina | Li & Brown, 2000 |
| Middle spotted woodpecker, Dendrocopos medius | Michalek & Winkler, 2001 |
| Monk parakeet, Myiopsitta monachus | Da Silva et al., 2010 |
| Montagu's harrier, Circus pygargus | Rutkowski et al., 2015 |
| Monteiros hornbill, Tockus monteiri | Stanback et al., 2002 |
| Nazca booby, Sula granti | Anderson & Boag, 2006 |
| New Zealand hihi, Notiomystis cincta | Brekke et al., 2013 |
| New Zealand robin, Petroica australis | Ardern et al., 1997; Taylor et al., 2008 |
| Noisy miner, Manorina melanocephala | Põldmaa et al., 1995 |
| North American black tern, Chlidonias niger | Shealer et al., 2014 |
| Northern bobwhite, Colinus virginianus | Davis et al., 2017 |
| Northern cardinal, Cardinalis cardinalis | Ritchison et al., 1994 |
| Northern flicker, Colaptes auratus | Wiebe & Kempenaers, 2009 |
| Northern wheatear, Oenanthe oenanthe | Currie et al., 1998, 1999 |
| Ostrich, Struthio camelus | Kimwele & Graves, 2002 |
| Palila, Loxioides bailleui | Fleischer et al., 1994 |
| Phainopepla, Phainopepla nitens | Chu et al., 2002 |
| Pied babbler, Turdoides bicolor | Nelson-Flower et al., 2011 |
| Pied flycatcher, Ficedula hypoleuca | Grinkov et al., 2018, 2020, 2022; Lifjeld et al., 1991; Moreno et al., 2013 |
| Prairie chicken, Tympanuchus cupido | Hess et al., 2012 |
| Pukeko, Porphyrio porphyrio | Jamieson et al., 1994 |
| Purple sandpiper, Calidris maritima | Pierce & Lifjeld, 1998 |
| Red phalarope, Phalaropus fulicarius | Dale et al., 1999 |
| Red-backed shrike, Lanius collurio | Fornasari et al., 1994 |
| Red-billed buffalo weaver, Bubalornis niger | Winterbottom et al., 2001 |
| Red-billed quelea, Quelea quelea | Dallimer & Jones, 2007 |
| Red-breasted flycatcher, Ficedula parva | Mitrus et al., 2014 |
| Red-capped plover, Charadrius ruficapillus | Maher et al., 2017 |
| Red-cockaded woodpecker, Picoides borealis | Haig et al., 1994 |
| Red-footed falcon, Falco vespertinus | Magonyi et al., 2021 |
| Red-necked phalarope, Phalaropus lobatus | Schamel et al., 2004 |
| Reed bunting, Emberiza schoeniclus | Bouwman et al., 2006 |
| Ridgway's hawk, Buteo ridgwayi | Woolaver et al., 2013 |
| Ringed plover, Charadrius hiaticula | Wallander et al., 2001 |
| Ross's goose, Chen rossii | Dunn et al., 1999 |
| Royal penguin, Eudyptes schlegeli | St Clair et al., 1995 |
| Ruff, Philomachus pugnax | Lank et al., 2002 |
| Rufous-and-white wren, Thryothorus rufalbus | Douglas et al., 2012 |
| Rufous-chested dotterel, Charadrius modestus | Maher et al., 2017 |
| Saddleback, Philesturnus carunculatus | Taylor et al., 2008 |
| Saffron finch, Sicalis flaveola | Benítez Saldívar et al., 2019 |
| Sanderling, Calidris alba | Reneerkens et al., 2014 |
| Savi's warbler, Locustella luscinioides | Neto et al., 2010 |
| Scaled quail, Callipepla squamata | Davis et al., 2017 |
| Scarlet rosefinch, Carpodacus erythrinus | Albrecht et al., 2007, 2009 |
| Scarlet tanager, Piranga olivacea | Klatt et al., 2008 |
| Scissor-tailed flycatchers, Tyrannus forficatus | Roeder et al., 2019 |
| Seaside sparrow, Ammodramus maritimus | Hill & Post, 2005 |
| Sedge warbler, Acrocephalus schoenobaenus | Langefors et al., 1998; Marshall et al., 2007 |
| Semipalmated plover, Charadrius semipalmatus | Zharikov & Nol, 2000 |
| Serin, Serinus serinus | Mota & Hoi-Leitner, 2003 |
| Siberian jay, Perisoreus infaustus | Gienapp & Merilä, 2010 |
| Smiths longspur, Calcarius pictus | Briskie et al., 1998 |
| Snowy plover, Charadrius nivosus | Maher et al., 2017 |
| Sociable weaver, Philetairus socius | Covas et al., 2006 |
| Song sparrow, Melospiza melodia | Major & Barber, 2004 |
| South polar skua, Catharacta maccormicki | Millar et al., 1997 |
| Southern emu-wren, Stipiturus malachurus | Maguire & Mulder, 2008 |
| Southern lapwing, Vanellus chilensis | Saracura et al., 2008 |
| Spotted towhee, Pipilo maculatus | Smith et al., 2016 |
| Stellers jay, Cyanocitta stelleri | Overeem et al., 2014 |
| Stripe-backed wren, Campylorhynchus nuchalis | Rabenold et al., 1990 |
| Subdesert mesite, Monias benschi | Seddon et al., 2005 |
| Superb fairy-wren, Malurus cyaneus | Colombelli-Négrel et al., 2009 |
| Swainsons hawk, Buteo swainsoni | Briggs & Collopy, 2012 |
| Takahe, Porphyrio hochstetteri | Lettink et al., 2002 |
| Tasmanian native hen, Gallinula mortierii | Lisle Gibbs et al., 1994 |
| Tawny owl, Strix aluco | Saladin et al., 2007 |
| Tree swallow, Tachycineta bicolor | Bitton et al., 2007; Kempenaers et al., 2001; O’Brien & Dawson, 2007; Stapleton et al., 2007 |
| Tufted titmouse, Baeolophus bicolor | Pravosudova et al., 2002 |
| Two-banded plover, Charadrius falklandicus | Maher et al., 2017 |
| Varied tit, Parus varius | Ju et al., 2014 |
| Vinous-throated parrotbills, Paradoxornis webbianus | Lee, 2012; Lee et al., 2009 |
| Western gull, Larus occidentalis | Gilbert et al., 1998 |
| Western sandpiper, Calidris mauri | Blomqvist et al., 2002 |
| Whiskered tern, Chlidonias hybrida | Ledwoń & Szczys, 2022 |
| White-crowned penduline tit, Remiz coronatus | Ball et al., 2017 |
| White-eyed vireo, Vireo griseus | Campomizzi et al., 2012 |
| White-fronted plover, Charadrius marginatus | Maher et al., 2017 |
| White-tailed ptarmigan, Lagopus leucura | Benson, 2002 |
| White-throated dipper, Cinclus cinclus | Øigarden et al., 2010 |
| White-winged chough, Corcorax melanorhamphos | Heinsohn et al., 2000 |
| Willow tit, Parus montanus | Orell et al., 1997 |
| Willow warbler, Phylloscopus trochilus | Gyllensten et al., 1990 |
| Wilson's phalarope, Steganopus tricolor | Delehanty et al., 1998 |
| Wood stork, Mycteria americana | Miño, Russello, et al., 2011 |
| Wood thrush, Hylocichla mustelina | Evans et al., 2008 |
| Wood warbler, Phylloscopus sibilatrix | Grendelmeier et al., 2017; Gyllensten et al., 1990 |
| Yellow rumped flycatcher, Ficedula zanthopygia | Mingju et al., 2017 |
| Yellow-breasted chat, Icteria virens | Miño, Pollet, et al., 2011 |
| Yellow-shouldered blackbird, Agelaius xanthomus | Liu et al., 2015 |
| Yellowhammer, Emberiza citrinella | Sundberg & Dixon, 1996 |
| Zebra finch, Taeniopygia guttata | Birkhead et al., 1990; Griffith et al., 2010 |

**References**

Alatalo, R. V., Burke, T., Dann, J., Hanotte, O., Hoglund, J., Lundberg, A., & Rintamaki, R. M. P. T. (1996). Paternity, copulation disturbance and female choice in lekking black grouse. *Anim. Behav*, *52*, 861–873.

Albrecht, T., Schnitzer, J., Kreisinger, J., Exnerova, A., Bryja, J., & Munclinger, P. (2007). Extrapair paternity and the opportunity for sexual selection in long-distant migratory passerines. *Behavioral Ecology*, *18*(2), 477–486. https://doi.org/10.1093/beheco/arm001

Albrecht, T., Vinkler, M., Schnitzer, J., Poláková, R., Munclinger, P., & Bryja, J. (2009). Extra-pair fertilizations contribute to selection on secondary male ornamentation in a socially monogamous passerine. *Journal of Evolutionary Biology*, *22*(10), 2020–2030. https://doi.org/10.1111/j.1420-9101.2009.01815.x

Alcaide, M., Negro, J. J., Serrano, D., Tella, J. L., & Rodríguez, C. (2005). Extra-pair paternity in the Lesser Kestrel Falco naumanni: A re-evaluation using microsatellite markers. *Ibis*, *147*(3), 608–611. https://doi.org/10.1111/j.1474-919x.2005.00429.x

Anderson, D. J., & Boag, P. T. (2006). No Extra-pair Fertilization Observed in Nazca Booby (Sula granti) Broods. *The Wilson Journal of Ornithology*, *118*(2), 244–247. https://doi.org/10.1676/05-106.1

Anker-Nilssen, Tycho. (2010). *Key-site monitoring in Røst in 2009* (No. 12–2010; SEAPOP ShortReport).

Ardern, S. L., Ewen, J. G., Armstrong, D. P., & Lambert, D. M. (1997). Social and Sexual Monogamy in Translocated New Zealand Robin Populations Detected Using Minisatellite DNA. *The Auk*, *114*(1), 120–126. https://doi.org/10.2307/4089074

Arsenault, D. P., Stacey, P. B., & Hoelzer, G. A. (2002). No Extra-Pair Fertilization in Flammulated Owls Despite Aggregated Nesting. *The Condor*, *104*(1), 197–202. https://doi.org/10.1093/condor/104.1.197

Ball, A. D., van Dijk, R. E., Lloyd, P., Pogány, Á., Dawson, D. A., Dorus, S., Bowie, R. C. K., Burke, T., & Székely, T. (2017). Levels of extra-pair paternity are associated with parental care in penduline tits (Remizidae). *Ibis*, *159*(2), 449–455. https://doi.org/10.1111/ibi.12446

Bao, X., Liu, F., Fan, Y., Jia, B., & Li, J. (2019). Extra-pair paternity in two passerine species living in an extreme-arid desert region. *The Wilson Journal of Ornithology*, *131*(3), 638. https://doi.org/10.1676/18-142

Benítez Saldívar, M. J., Miño, C. I., & Massoni, V. (2019). Genetic mating system, population genetics and effective size of Saffron Finches breeding in southern South America. *Genetica*, *147*(3–4), 315–326. https://doi.org/10.1007/s10709-019-00072-4

Benson, D. P. (2002). Low Extra-Pair Paternity in White-Tailed Ptarmigan. *The Condor*, *104*(1), 192–197. https://doi.org/10.1093/condor/104.1.192

Bird, K. L., Aldridge, C. L., Carpenter, J. E., Paszkowski, C. A., Boyce, M. S., & Coltman, D. W. (2013). The secret sex lives of sage-grouse: Multiple paternity and intraspecific nest parasitism revealed through genetic analysis. *Behavioral Ecology*, *24*(1), 29–38. https://doi.org/10.1093/beheco/ars132

Birkhead, T. R., Burke, T., Zann, R., Hunter, F. M., & Krupa, A. P. (1990). Extra-pair paternity and intraspecific brood parasitism in wild zebra finches Taeniopygia guttata, revealed by DNA fingerprinting. *Behavioral Ecology and Sociobiology*, *27*(5), 315–324. https://doi.org/10.1007/BF00164002

Bitton, P.-P., O’Brien, E. L., & Dawson, R. D. (2007). Plumage brightness and age predict extrapair fertilization success of male tree swallows, Tachycineta bicolor. *Animal Behaviour*, *74*(6), 1777–1784. https://doi.org/10.1016/j.anbehav.2007.03.018

Blomqvist, D., Andersson, M., Küpper, C., Cuthill, I. C., Kis, J., Lanctot, R. B., Sandercock, B. K., Székely, T., Wallander, J., & Kempenaers, B. (2002). Genetic similarity between mates and extra-pair parentage in three species of shorebirds. *Nature*, *419*(6907), 613–615. https://doi.org/10.1038/nature01104

Bønløkke-Pedersen, J., Drachmann, J., Frydenberg, J., & Boomsma, J. J. (2002). Rare extra-pair fertilizations in the semi-colonially breeding linnet Carduelis cannabina. *Journal of Avian Biology*, *33*(2), 203–206. https://doi.org/10.1034/j.1600-048X.2002.330213.x

Bouwman, K. M., Burke, T., & Komdeur, J. (2006). How female reed buntings benefit from extra-pair mating behaviour: Testing hypotheses through patterns of paternity in sequential broods. *Molecular Ecology*, *15*(9), 2589–2600. https://doi.org/10.1111/j.1365-294X.2006.02955.x

Brekke, P., Cassey, P., Ariani, C., & Ewen, J. G. (2013). Evolution of extreme-mating behaviour: Patterns of extrapair paternity in a species with forced extrapair copulation. *Behavioral Ecology and Sociobiology*, *67*(6), 963–972. https://doi.org/10.1007/s00265-013-1522-9

Briggs, C. W., & Collopy, M. W. (2012). Extra-pair paternity in Swainson’s Hawks: Swainson’s Hawk Extra-Pair Paternity. *Journal of Field Ornithology*, *83*(1), 41–46. https://doi.org/10.1111/j.1557-9263.2011.00354.x

Briskie, J. V., Montgomerie, R., Boag, P. T., & Põldmaa, T. (1998). Paternity and paternal care in the polygynandrous Smith’s longspur. *Behavioral Ecology and Sociobiology*, *43*(3), 181–190. https://doi.org/10.1007/s002650050479

Bruce, J. P., Quinn, J. S., Sloane, S. A., & White, B. N. (1996). DNA Fingerprinting Reveals Monogamy in the Bushtit, a Cooperatively Breeding Species. *The Auk*, *113*(2), 511–516. https://doi.org/10.2307/4088921

Brylawski, A. M. Z., & Whittingham, L. A. (2004). An experimental study of mate guarding and paternity in house wrens. *Animal Behaviour*, *68*(6), 1417–1424. https://doi.org/10.1016/j.anbehav.2004.02.016

Bukacińska, M., Bukaciński, D., Epplen, J. T., Sauer, K. P., & Lubjuhn, T. (1998). Low frequency of extra-pair paternity in Common Gulls (Larus canus) as revealed by DNA fingerprinting. *Journal Für Ornithologie*, *139*(4), 413–420. https://doi.org/10.1007/BF01653468

Burke, T., Davies, N. B., Bruford, M. W., & Hatchwell, B. J. (1989). Parental care and mating behaviour of polyandrous dunnocks Prunella modularis related to paternity by DNA fingerprinting. *Nature*, *338*(6212), 249–251. https://doi.org/10.1038/338249a0

Byers, B. E., Mays, H. L., Stewart, I. R. K., & Westneat, D. F. (2004). Extrapair paternity increases variability in male reproductive success in the chestnut-slded warbler (Dendroica pensylvanica), a socially monogamous songbird. *The Auk*, *121*(3), 788. https://doi.org/10.1642/0004-8038(2004)121[0788:EPIVIM]2.0.CO;2

Calderón, L., Svagelj, W. S., Quintana, F., Lougheed, S. C., & Tubaro, P. L. (2012). No evidence of extra-pair paternity or intraspecific brood parasitism in the Imperial Shag Phalacrocorax atriceps. *Journal of Ornithology*, *153*(2), 399–404. https://doi.org/10.1007/s10336-011-0754-6

Campomizzi, A. J., Morrison, M. L., DeWoody, J. A., Farrell, S. L., & Wilkins, R. N. (2012). Win-stay, lose-switch and public information strategies for patch fidelity of songbirds with rare extra-pair paternity. *Scientific Reports*, *2*(1), 294. https://doi.org/10.1038/srep00294

Carvalho, C. B. V., Macedo, R. H., & Graves, J. A. (2006). Breeding Strategies of a Socially Monogamous Neotropical Passerine: Extra-Pair Fertilizations, Behavior, and Morphology. *The Condor*, *108*(3), 579–590. https://doi.org/10.1093/condor/108.3.579

Celis-Murillo, A., Schelsky, W., Benson, T. J., Louder, M. I. M., & Ward, M. P. (2017). Patterns, correlates, and paternity consequences of extraterritorial foray behavior in the field sparrow (Spizella pusilla): An automated telemetry approach. *Behavioral Ecology and Sociobiology*, *71*(2), 45. https://doi.org/10.1007/s00265-017-2273-9

Choudhury, S., Jones, C. S., Black, J. M., & Prop, J. (1993). Adoption of Young and Intraspecific Nest Parasitism in Barnacle Geese. *The Condor*, *95*(4), 860–868. https://doi.org/10.2307/1369423

Chu, M., Koenig, W. D., Godinez, A., McIntosh, C. E., & Fleischer, R. C. (2002). Social and Genetic Monogamy in Territorial and Loosely Colonial Populations of Phainopepla (Phainopepla Nitens). *The Auk*, *119*(3), 770–777. https://doi.org/10.1093/auk/119.3.770

Colombelli-Négrel, D., Schlotfeldt, B. E., & Kleindorfer, S. (2009). High levels of extra-pair paternity in Superb Fairy-wrens in South Australia despite low frequency of auxiliary males. *Emu - Austral Ornithology*, *109*(4), 300–304. https://doi.org/10.1071/MU09035

Conrad, K. F., Clarke, M. F., Robertson, R. J., & Boag, P. T. (1998). Paternity and the Relatedness of Helpers in the Cooperatively Breeding Bell Miner. *The Condor*, *100*(2), 343–349. https://doi.org/10.2307/1370275

Cordero, P. J., Wetton, J. H., & Parkin, D. T. (1999). Extra-Pair Paternity and Male Badge Size in the House Sparrow. *Journal of Avian Biology*, *30*(1), 97. https://doi.org/10.2307/3677248

Covas, R., Dalecky, A., Caizergues, A., & Doutrelant, C. (2006). Kin associations and direct vs indirect fitness benefits in colonial cooperatively breeding sociable weavers Philetairus socius. *Behavioral Ecology and Sociobiology*, *60*(3), 323–331. https://doi.org/10.1007/s00265-006-0168-2

Cramer, E. R. A., Hall, M. L., de Kort, S. R., Lovette, I. J., & Vehrencamp, S. L. (2011). Infrequent Extra-Pair Paternity in the Banded Wren, a Synchronously Breeding Tropical Passerine. *The Condor*, *113*(3), 637–645. https://doi.org/10.1525/cond.2011.100233

Cramer, E. R. A., Laskemoen, T., Kleven, O., LaBarbera, K., Lovette, I. J., & Lifjeld, J. T. (2013). No evidence that sperm morphology predicts paternity success in wild house wrens. *Behavioral Ecology and Sociobiology*, *67*(11), 1845–1853. https://doi.org/10.1007/s00265-013-1594-6

Currie, D. R., Burke, T., Whitney, R. L., & Thompson, D. B. A. (1998). Male and female behaviour and extra-pair paternity in the wheatear. *Animal Behaviour*, *55*(3), 689–703. https://doi.org/10.1006/anbe.1997.0750

Currie, D. R., Krupa, A. P., Burke, T., & Thompson, D. B. A. (1999). The effect of experimental male removals on extrapair paternity in the wheatear,Oenanthe oenanthe. *Animal Behaviour*, *57*(1), 145–152. https://doi.org/10.1006/anbe.1998.0960

Da Silva, A. G., Eberhard, J. R., Wright, T. F., Avery, M. L., & Russello, M. A. (2010). Genetic evidence for high propagule pressure and long-distance dispersal in monk parakeet (Myiopsitta monachus) invasive populations. *Molecular Ecology*, *19*(16), 3336–3350. https://doi.org/10.1111/j.1365-294X.2010.04749.x

Dale, J., Montgomerie, R., Michaud, D., & Boag, P. (1999). Frequency and timing of extrapair fertilisation in the polyandrous red phalarope (Phalaropus fulicarius). *Behavioral Ecology and Sociobiology*, *46*(1), 50–56. https://doi.org/10.1007/s002650050591

Dallimer, M., & Jones, P. J. (2007). An estimation of the rate of reproductive cheating in the Red-billed Quelea *Quelea quelea*. *Ostrich*, *78*(3), 637–639. https://doi.org/10.2989/OSTRICH.2007.78.3.11.322

Davies, N. B., Butchart, S. H. M., Burke, T. A., Chaline, N., & Stewart, I. R. K. (2003). Reed warblers guard against cuckoos and cuckoldry. *Animal Behaviour*, *65*(2), 285–295. https://doi.org/10.1006/anbe.2003.2049

Davis, C. A., Orange, J. P., Van Den Bussche, R. A., Elmore, R. D., Fuhlendorf, S. D., Carroll, J. M., Tanner, E. P., & Leslie, D. M. (2017). Extrapair paternity and nest parasitism in two sympatric quail. *The Auk*, *134*(4), 811–820. https://doi.org/10.1642/AUK-16-162.1

Decker, M. D., Parker, P. G., Minchella, D. J., & Rabenold, K. N. (1993). Monogamy in black vultures: Genetic evidence from DNA fingerprinting. *Behavioral Ecology*, *4*(1), 29–35. https://doi.org/10.1093/beheco/4.1.29

Delehanty, D. J., Fleischer, R. C., Colwell, M. A., & Oring, L. W. (1998). Sex-role reversal and the absence of extra-pair fertilization in Wilson’s phalaropes. *Animal Behaviour*, *55*(4), 995–1002. https://doi.org/10.1006/anbe.1997.0670

Dias, R. I., Macedo, R. H., Goedert, D., & Webster, M. S. (2013). Cooperative Breeding in the Campo Flicker II: Patterns of Reproduction and Kinship. *The Condor*, *115*(4), 855–862. https://doi.org/10.1525/cond.2013.120143

Dickinson, J., Haydock, J., Koenig, W., Stanback, M., & Pitelka, F. (1995). Genetic monogamy in single-male groups of acorn woodpeckers, Melanerpes formicivorus. *Molecular Ecology*, *4*(6), 765–770. https://doi.org/10.1111/j.1365-294X.1995.tb00277.x

Douglas, S. B., Heath, D. D., & Mennill, D. J. (2012). Low Levels of Extra-Pair Paternity in a Neotropical Duetting Songbird, the Rufous-and-white Wren (*Thryothorus rufalbus*). *The Condor*, *114*(2), 393–400. https://doi.org/10.1525/cond.2012.110028

Dunn, P. O., Afton, A. D., Gloutney, M. L., & Alisauskas, R. T. (1999). Forced copulation results in few extrapair fertilizations in Ross’s and lesser snow geese. *Animal Behaviour*, *57*(5), 1071–1081. https://doi.org/10.1006/anbe.1998.1066

Eastwood, J. R., Berg, M. L., Ribot, R. F. H., Stokes, H. S., Martens, J. M., Buchanan, K. L., Walder, K., & Bennett, A. T. D. (2018). Pair fidelity in long-lived parrots: Genetic and behavioural evidence from the Crimson Rosella (*Platycercus elegans*). *Emu - Austral Ornithology*, *118*(4), 369–374. https://doi.org/10.1080/01584197.2018.1453304

Evans, M. L., Stutchbury, B. J. M., & Woolfenden, B. E. (2008). Off-Territory Forays and Genetic Mating System of the Wood Thrush (*Hylocichla mustelina*). *The Auk*, *125*(1), 67–75. https://doi.org/10.1525/auk.2008.125.1.67

Evans, M. L., Woolfenden, B. E., Friesen, L., & Stutchbury, B. J. M. (2009). Variation in the extra-pair mating systems of Acadian Flycatchers and Wood Thrushes in forest fragments in southern Ontario. *Journal of Field Ornithology*, *80*(2), 146–153. https://doi.org/10.1111/j.1557-9263.2009.00216.x

Faaborg, J., Parker, P. G., DeLay, L., de Vries, Tj., Bednarz, J. C., Maria Paz, S., Naranjo, J., & Waite, T. A. (1995). Confirmation of cooperative polyandry in the Galapagos hawk (Buteo galapagoensis). *Behavioral Ecology and Sociobiology*, *36*(2), 83–90. https://doi.org/10.1007/BF00170712

Ferretti, V., Liljesthröm, M., López, A. S., Lovette, I. J., & Winkler, D. W. (2016). Extra‐pair paternity in a population of Chilean Swallows breeding at 54 degrees south. *Journal of Field Ornithology*, *87*(2), 155–161. https://doi.org/10.1111/jofo.12144

Fleischer, R. C., Tarr, C. L., Morton, E. S., Sangmeister, A., & Derrickson, K. C. (1997). Mating System of the Dusky Antbird, a Tropical Passerine, as Assessed by DNA Fingerprinting. *The Condor*, *99*(2), 512–514. https://doi.org/10.2307/1369957

Fleischer, R. C., Tarr, C. L., & Pratt, T. K. (1994). Genetic structure and mating system in the palila, an endangered Hawaiian honeycreeper, as assessed by DNA fingerprinting. *Molecular Ecology*, *3*(4), 383–392. https://doi.org/10.1111/j.1365-294X.1994.tb00078.x

Fornasari, L., Bottoni, L., Sacchi, N., & Massa, R. (1994). Home range overlapping and socio-sexual relationships in the red-backed shrike *Lanius collurio*. *Ethology Ecology & Evolution*, *6*(2), 169–177. https://doi.org/10.1080/08927014.1994.9522992

Forslund, P., & Larsson, K. (1995). Intraspecific nest parasitism in the barnacle goose: Behavioural tactics of parasites and hosts. *Animal Behaviour*, *50*(2), 509–517. https://doi.org/10.1006/anbe.1995.0265

García-Navas, V., Ferrer, E. S., Cáliz-Campal, C., Bueno-Enciso, J., Barrientos, R., Sanz, J. J., & Ortego, J. (2015). Spatiotemporal and genetic contingency of extrapair behaviour in a songbird. *Animal Behaviour*, *106*, 157–169. https://doi.org/10.1016/j.anbehav.2015.05.020

Gienapp, P., & Merilä, J. (2010). High Fidelity – No Evidence for Extra-Pair Paternity in Siberian Jays (Perisoreus infaustus). *PLoS ONE*, *5*(8), e12006. https://doi.org/10.1371/journal.pone.0012006

Gilbert, L., Burke, T., & Krupa, A. (1998). No evidence for extra‐pair paternity in the western gull. *Molecular Ecology*, *7*(11), 1549–1552. https://doi.org/10.1046/j.1365-294x.1998.00488.x

Gill, S. A., Vonhof, M. J., Stutchbury, B. J. M., Morton, E. S., & Quinn, J. S. (2005). No evidence for acoustic mate-guarding in duetting buff-breasted wrens (Thryothorus leucotis). *Behavioral Ecology and Sociobiology*, *57*(6), 557–565. https://doi.org/10.1007/s00265-004-0893-3

González-Solı́s, J., Sokolov, E., & Becker, P. H. (2001). Courtship feedings, copulations and paternity in common terns, Sterna hirundo. *Animal Behaviour*, *61*(6), 1125–1132. https://doi.org/10.1006/anbe.2001.1711

Green, D. J., Peters, A., & Cockburn, A. (2002). Extra-pair paternity and mate-guarding behaviour in the brown thornbill. *Australian Journal of Zoology*, *50*(6), 565. https://doi.org/10.1071/ZO02037

Grendelmeier, A., Arlettaz, R., Olano-Marin, J., & Pasinelli, G. (2017). Experimentally provided conspecific cues boost bird territory density but not breeding performance. *Behavioral Ecology*, *28*(1), 174–185. https://doi.org/10.1093/beheco/arw144

Griffith, S. C., Holleley, C. E., Mariette, M. M., Pryke, S. R., & Svedin, N. (2010). Low level of extrapair parentage in wild zebra finches. *Animal Behaviour*, *79*(2), 261–264. https://doi.org/10.1016/j.anbehav.2009.11.031

Griffith, S. C., Stewart, I. R. K., Dawson, D. A., Owens, I. P. F., & Burke, T. (1999). Contrasting levels of extra-pair paternity in mainland and island populations of the house sparrow (Passer domesticus): Is there an ‘island effect’? *Biological Journal of the Linnean Society*, *68*(1–2), 303–316. https://doi.org/10.1111/j.1095-8312.1999.tb01171.x

Griggio, M., Matessi, G., & Marin, G. (2004). No evidence of extra‐pair paternity in a colonial seabird, the common tern *(Sterna hirundo)*. *Italian Journal of Zoology*, *71*(3), 219–222. https://doi.org/10.1080/11250000409356575

Grinkov, V. G., Bauer, A., Gashkov, S. I., Sternberg, H., & Wink, M. (2018). Diversity of social-genetic relationships in the socially monogamous pied flycatcher (*Ficedula hypoleuca*) breeding in Western Siberia. *PeerJ*, *6*, e6059. https://doi.org/10.7717/peerj.6059

Grinkov, V. G., Bauer, A., Sternberg, H., & Wink, M. (2020). Heritability of the extra-pair mating behaviour of the pied flycatcher in Western Siberia. *PeerJ*, *8*, e9571. https://doi.org/10.7717/peerj.9571

Grinkov, V. G., Bauer, A., Sternberg, H., & Wink, M. (2022). Understanding Extra-Pair Mating Behaviour: A Case Study of Socially Monogamous European Pied Flycatcher (Ficedula hypoleuca) in Western Siberia. *Diversity*, *14*(4), 283. https://doi.org/10.3390/d14040283

Gyllensten, U. B., Jakobsson, S., & Temrin, H. (1990). No evidence for illegitimate young in monogamous and polygynous warblers. *Nature*, *343*(6254), 168–170. https://doi.org/10.1038/343168a0

Haggerty, T. M., Morton, E. S., & Fleischer, R. C. (2001). Genetic Monogamy in Carolina Wrens (Thryothorus ludovicianus). *The Auk*, *118*(1), 215–219. https://doi.org/10.1093/auk/118.1.215

Haig, S. M. (2003). Parentage and Relatedness in Polyandrous Comb-Crested Jacanas Using ISSRs. *Journal of Heredity*, *94*(4), 302–309. https://doi.org/10.1093/jhered/esg072

Haig, S. M., Walters, J. R., & Plissner, J. H. (1994). Genetic evidence for monogamy in the cooperatively breeding red-cockaded woodpecker. *Behavioral Ecology and Sociobiology*, *34*(4), 295–303. https://doi.org/10.1007/BF00183480

Hall, M. L., & Magrath, R. D. (2000). Duetting and mate-guarding in Australian magpie-larks ( Grallina cyanoleuca). *Behavioral Ecology and Sociobiology*, *47*(3), 180–187. https://doi.org/10.1007/s002650050009

Hamao, S., & Saito, D. S. (2005). Extrapair Fertilization in the Black-Browed Reed Warbler (Acrocephalus Bistrigiceps): Effects on Mating Status and Nesting Cycle of Cuckolded and Cuckolder Males. *The Auk*, *122*(4), 1086–1096. https://doi.org/10.1093/auk/122.4.1086

Hansson, B., Hasselquist, D., & Bensch, S. (2004). Do female great reed warblers seek extra–pair fertilizations to avoid inbreeding? *Proceedings of the Royal Society of London. Series B: Biological Sciences*, *271*(suppl_5). https://doi.org/10.1098/rsbl.2004.0164

Hartley, I. R., Davies, N. B., Hatchwell, B. J., Desrochers, A., Nebel, D., & Burke, T. (1995). The polygynandrous mating system of the alpine accentor, Prunella collaris. II. Multiple paternity and parental effort. *Animal Behaviour*, *49*(3), 789–803. https://doi.org/10.1016/0003-3472(95)80210-X

Hartley, I. R., Shepherd, M., & Burke, T. R. T. (1993). Reproductive success of polygynous male corn buntings (*Miliaria calandra*) as confirmed by DNA fingerprinting. *Behavioral Ecology*, *4*(4), 310–317. https://doi.org/10.1093/beheco/4.4.310

Hasselquist, D., Bensch, S., & von Schantz, T. (1996). Correlation between male song repertoire, extra-pair paternity and offspring survival in the great reed warbler. *Nature*, *381*(6579), 229–232. https://doi.org/10.1038/381229a0

Hatchwell, B. J., Ross, D. J., Chaline, N., Fowlie, M. K., & Burke, T. (2002). Parentage in the cooperative breeding system of long-tailed tits, Aegithalos caudatus. *Animal Behaviour*, *64*(1), 55–63. https://doi.org/10.1006/anbe.2002.3033

Heer, L. (1996). Cooperative breeding by Alpine AccentorsPrunella collaris: Polygynandry, territoriality and multiple paternity. *Journal Für Ornithologie*, *137*(1), 35–51. https://doi.org/10.1007/BF01651498

Heg, D., Ens, B. J., Burke, T., Jenkins, L., & Kruijt, J. P. (1993). Why Does the Typically Monogamous Oystercatcher (Haematopus Ostralegus) Engage in Extra-Pair Copulations? *Behaviour*, *126*(3–4), 247–289. https://doi.org/10.1163/156853993X00137

Heinsohn, R., Dunn, P., Legge, S., & Double, M. (2000). Coalitions of relatives and reproductive skew in cooperatively breeding white-winged choughs. *Proceedings of the Royal Society of London. Series B: Biological Sciences*, *267*(1440), 243–249. https://doi.org/10.1098/rspb.2000.0993

Helfenstein, F., Tirard, C., Danchin, E., & Wagner, R. H. (2004). Low Frequency of Extra-Pair Paternity and High Frequency of Adoption in Black-Legged Kittiwakes. *The Condor*, *106*, 149–155. https://doi.org/10.1093/condor/106.1.149

Henderson, I. G., Hart, P. J. B., & Burke, T. (2000). Strict monogamy in a semi-colonial passerine: The Jackdaw *Corvus monedula*. *Journal of Avian Biology*, *31*(2), 177–182. https://doi.org/10.1034/j.1600-048X.2000.310209.x

Henry, I., Antoniazza, S., Dubey, S., Simon, C., Waldvogel, C., Burri, R., & Roulin, A. (2013). Multiple Paternity in Polyandrous Barn Owls (Tyto alba). *PLoS ONE*, *8*(11), e80112. https://doi.org/10.1371/journal.pone.0080112

Hess, B. D., Dunn, P. O., & Whittingham, L. A. (2012). Females choose multiple mates in the lekking Greater Prairie-Chicken (*Tympanuchus cupido*). *The Auk*, *129*(1), 133–139. https://doi.org/10.1525/auk.2011.11095

Hill, C. E., & Post, W. (2005). Extra-pair paternity in Seaside Sparrows. *Journal of Field Ornithology*, *76*(2), 119–126. https://doi.org/10.1648/0273-8570-76.2.119

Hoffman, J. I., Munro, K., Kilner, R. M., & Amos, W. (2010). High rates of infidelity in the Grey Fantail *Rhipidura albiscapa* suggest that testis size may be a better correlate of extra-pair paternity than sexual dimorphism. *Ibis*, *152*(2), 378–385. https://doi.org/10.1111/j.1474-919X.2009.01007.x

Hsu, Y.-C., Li, S.-H., Lin, Y.-S., Philippart, M. T., & Severinghaus, L. L. (2006). High frequency of extrapair copulation with low level of extrapair fertilization in the Lanyu scops owl *Otus elegans botelensis*. *Journal of Avian Biology*, *37*(1), 36–40. https://doi.org/10.1111/j.2006.0908-8857.03687.x

Jamieson, I. G., Quinn, J. S., Rose, P. A., & White, B. N. (1994). Shared paternity among non-relatives is a result of an egalitarian mating system in a communally breeding bird, the pukeko. *Proceedings of the Royal Society of London. Series B: Biological Sciences*, *257*(1350), 271–277. https://doi.org/10.1098/rspb.1994.0125

Johannessen, L. E., Ke, D., Lu, X., & Lifjeld, J. T. (2011). Geographical variation in patterns of parentage and relatedness in the co-operatively breeding Ground Tit Parus humilis. *Ibis*, *153*(2), 373–383. https://doi.org/10.1111/j.1474-919X.2011.01115.x

Johannessen, L. E., Slagsvold, T., Hansen, B. T., & Lifjeld, J. T. (2005). Manipulation of male quality in wild tits: Effects on paternity loss. *Behavioral Ecology*, *16*(4), 747–754. https://doi.org/10.1093/beheco/ari048

Ju, J., Yin, J., Racey, P., Zhang, L., Li, D., & Wan, D. (2014). Extra-Pair Paternity in Varied Tits *Poecile varius*. *Acta Ornithologica*, *49*(1), 131–137. https://doi.org/10.3161/000164514X682959

Kempenaers, B., Everding, S., Bishop, C., Boag, P., & Robertson, R. J. (2001). Extra-pair paternity and the reproductive role of male floaters in the tree swallow (Tachycineta bicolor). *Behavioral Ecology and Sociobiology*, *49*(4), 251–259. https://doi.org/10.1007/s002650000305

Kimwele, C. N., & Graves, J. A. (2002). A molecular genetic analysis of the communal nesting of the ostrich (Struthio camelus). *Molecular Ecology*, *12*(1), 229–236. https://doi.org/10.1046/j.1365-294X.2003.01727.x

Klatt, P. H., Stutchbury, B. J. M., & Evans, M. L. (2008). Incubation feeding by male Scarlet Tanagers: A mate removal experiment. *Journal of Field Ornithology*, *79*(1), 1–10. https://doi.org/10.1111/j.1557-9263.2008.00139.x

Klauke, N., Segelbacher, G., & Schaefer, H. M. (2013). Reproductive success depends on the quality of helpers in the endangered, cooperative El Oro parakeet (*Pyrrhura orcesi*). *Molecular Ecology*, *22*(7), 2011–2027. https://doi.org/10.1111/mec.12219

Kleven, O., Bjerke, B.-A., & Lifjeld, J. T. (2008). Genetic monogamy in the Common Crossbill (Loxia curvirostra). *Journal of Ornithology*, *149*(4), 651–654. https://doi.org/10.1007/s10336-008-0291-0

Kleven, O., Øigarden, T., Foyn, B. E., Moksnes, A., Røskaft, E., Rudolfsen, G., Stokke, B. G., & Lifjeld, J. T. (2007). Low frequency of extrapair paternity in the common redstart (Phoenicurus phoenicurus). *Journal of Ornithology*, *148*(3), 373–378. https://doi.org/10.1007/s10336-007-0139-z

Koopman, M. E., McDonald, D. B., & Hayward, G. D. (2007). Microsatellite Analysis Reveals Genetic Monogamy Among Female Boreal Owls. *Journal of Raptor Research*, *41*(4), 314–318. https://doi.org/10.3356/0892-1016(2007)41[314:MARGMA]2.0.CO;2

Korpimäki, E., Lahti, K., May, C. A., Parkin, D. T., Powell, G. B., Tolonen, P., & Wetton, J. H. (1996). Copulatory behaviour and paternity determined by DNA fingerprinting in kestrels: Effects of cyclic food abundance. *Animal Behaviour*, *51*(4), 945–955. https://doi.org/10.1006/anbe.1996.0098

Kreisinger, J., Munclinger, P., Javůrková, V., & Albrecht, T. (2010). Analysis of extra-pair paternity and conspecific brood parasitism in mallards Anas platyrhynchos using non-invasive techniques. *Journal of Avian Biology*, *41*(5), 551–557. https://doi.org/10.1111/j.1600-048X.2010.05002.x

Krokene, C. (1998). The function of extrapair paternity in blue tits and great tits: Good genes or fertility insurance? *Behavioral Ecology*, *9*(6), 649–656. https://doi.org/10.1093/beheco/9.6.649

Krueger, T. R., Williams, D. A., & Searcy, W. A. (2008). The genetic mating system of a tropical tanager. *The Condor*, *110*(3), 559–562. https://doi.org/10.1525/cond.2008.8546

Langefors, A., Hasselquist, D., & von Schantz, T. (1998). Extra-Pair Fertilizations in the Sedge Warbler. *Journal of Avian Biology*, *29*(2), 134. https://doi.org/10.2307/3677191

Lank, D. B., Smith, C. M., Hanotte, O., Ohtonen, A., Bailey, S., & Burke, T. (2002). High frequency of polyandry in a lek mating system. *Behavioral Ecology*, *13*(2), 209–215. https://doi.org/10.1093/beheco/13.2.209

Larsson, K., Tegelström, H., & Forslund, P. (1995). Intraspecific nest parasitism and adoption of young in the barnaele goose: Effects on survival and reproductive performance. *Animal Behaviour*, *50*(5), 1349–1360. https://doi.org/10.1016/0003-3472(95)80050-6

Lawless, S. G., Ritchison, G., Klatt, P. H., & Westneat, D. F. (1997). The Mating Strategies of Eastern Screech-Owls: A Genetic Analysis. *The Condor*, *99*(1), 213–217. https://doi.org/10.2307/1370242

Lebigre, C., Alatalo, R. V., Siitari, H., & Parri, S. (2007). Restrictive mating by females on black grouse leks. *Molecular Ecology*, *16*(20), 4380–4389. https://doi.org/10.1111/j.1365-294X.2007.03502.x

Ledwoń, M., & Szczys, P. (2022). Extra-pair paternity in a species with frequent extra-pair courtship feedings, few extra-pair copulations, and male-biased parental care. *Journal of Ornithology*, *163*(2), 437–444. https://doi.org/10.1007/s10336-021-01954-1

Lee, J.-W. (2012). Females may not obtain indirect genetic benefits from extra-pair paternity in vinous-throated parrotbills, Paradoxornis webbianus. *Journal of Ethology*, *30*(1), 53–59. https://doi.org/10.1007/s10164-011-0292-y

Lee, J.-W., Kim, M.-S., Burke, T., & Hatchwell, B. J. (2009). Extrapair paternity in a flock-living passerine, the vinous-throated parrotbill *Paradoxornis webbianus*. *Journal of Avian Biology*, *40*(5), 469–474. https://doi.org/10.1111/j.1600-048X.2009.04836.x

Legge, S., & Cockburn, A. (2000). Social and mating system of cooperatively breeding laughing kookaburras (Dacelo novaeguineae). *Behavioral Ecology and Sociobiology*, *47*(4), 220–229. https://doi.org/10.1007/s002650050659

Leisler, B., Beier, J., Staudter, H., & Wink, M. (2001). Variation in extra-pair paternity in the polygynous Great Reed Warbler (Acrocephalus arundinaceus). *Journal Für Ornithologie*, *141*(1), 77. https://doi.org/10.1046/j.1439-0361.2000.00047.x

Leitão, A. V., Hall, M. L., & Mulder, R. A. (2021). Female and male plumage color is linked to parental quality, pairing, and extrapair mating in a tropical passerine. *Behavioral Ecology*, *32*(3), 452–463. https://doi.org/10.1093/beheco/araa154

Lettink, M., Jamieson, I. G., Millar, C. D., & Lambert, D. M. (2002). Mating system and genetic variation in the endangered New Zealand takahe. *Conservation Genetics*, *3*(4), 427–434. https://doi.org/10.1023/A:1020567701633

Li, M.-H., Välimäki, K., Piha, M., Pakkala, T., & Merilä, J. (2009). Extrapair paternity and maternity in the three-toed woodpecker, Picoides tridactylus: Insights from microsatellite-based parentage analysis. *PLoS ONE*, *4*(11), e7895. https://doi.org/10.1371/journal.pone.0007895

Li, S.-H., & Brown, J. L. (2000). High frequency of extrapair fertilization in a plural breeding bird, the Mexican jay, revealed by DNA microsatellites. *Animal Behaviour*, *60*(6), 867–877. https://doi.org/10.1006/anbe.2000.1554

Liebers, D., & Peter, H.-U. (1998). Intraspecific interactions in jackdaws *Corvus monedula*: A field study combined with parentage analysis. *Ardea*, *86*, 221–235.

Lifjeld, J. T., Slagsvold, T., & Lampe, H. M. (1991). Low frequency of extra-pair paternity in pied flycatchers revealed by DNA fingerprinting. *Behavioral Ecology and Sociobiology*, *29*(2), 95–101. https://doi.org/10.1007/BF00166483

Lindstedt, E. R., Oh, K. P., & Badyaev, A. V. (2007). Ecological, social, and genetic contingency of extrapair behavior in a socially monogamous bird. *Journal of Avian Biology*, *38*(2), 214–223. https://doi.org/10.1111/j.2007.0908-8857.03889.x

Lisle Gibbs, H., Goldizen, A. W., Bullough, C., & Goldizen, A. R. (1994). Parentage analysis of multi-male social groups of tasmanian native hens (Tribonyx mortierii): Genetic evidence for monogamy and polyandry. *Behavioral Ecology and Sociobiology*, *35*(5), 363–371. https://doi.org/10.1007/BF00184425

Liu, I. A., Johndrow, J. E., Abe, J., Lüpold, S., Yasukawa, K., Westneat, D. F., & Nowicki, S. (2015). Genetic diversity does not explain variation in extra-pair paternity in multiple populations of a songbird. *Journal of Evolutionary Biology*, *28*(5), 1156–1169. https://doi.org/10.1111/jeb.12644

Lopes, I. F., Miño, C. I., Rocha, C. D., Oliveira, D. M. M., & Del Lama, S. N. (2013). Inferred kinship patterns reveal low levels of extra-pair paternity in the endangered Neotropical Jabiru Stork (Jabiru mycteria, Aves: Ciconiiformes). *Genetica*, *141*(4–6), 195–203. https://doi.org/10.1007/s10709-013-9718-5

Lundy, K. J., Parker, P. G., & Zahavi, A. (1998). Reproduction by subordinates in cooperatively breeding Arabian babblers is uncommon but predictable. *Behavioral Ecology and Sociobiology*, *43*(3), 173–180. https://doi.org/10.1007/s002650050478

Magonyi, N. M., Szabó, K., Fehérvári, P., Solt, S., Palatitz, P., Vili, N., Bertók, P., & Mátics, R. (2021). Extra‐pair paternity, intraspecific brood parasitism, quasi‐parasitism and polygamy in the Red‐footed Falcon (*Falco vespertinus*). *Ibis*, *163*(3), 1087–1092. https://doi.org/10.1111/ibi.12932

Maguire, G. S., & Mulder, R. A. (2008). Low levels of extra-pair paternity in southern emu-wrens (Aves: Maluridae). *Australian Journal of Zoology*, *56*(2), 79. https://doi.org/10.1071/ZO08047

Maher, K. H., Eberhart-Phillips, L. J., Kosztolányi, A., Remedios, N. dos, Carmona-Isunza, M. C., Cruz-López, M., Zefania, S., St Clair, J. J. H., Alrashidi, M., Weston, M. A., Serrano-Meneses, M. A., Krüger, O., Hoffman, J. I., Székely, T., Burke, T., & Küpper, C. (2017). High fidelity: Extra-pair fertilisations in eight *Charadrius* plover species are not associated with parental relatedness or social mating system. *Journal of Avian Biology*, *48*(7), 910–920. https://doi.org/10.1111/jav.01263

Major, D. L., & Barber, C. A. (2004). Extra-pair paternity in first and second broods of eastern Song Sparrows. *Journal of Field Ornithology*, *75*(2), 152–156. https://doi.org/10.1648/0273-8570-75.2.152

Marks, J. S., Dickinson, J. L., & Haydock, J. (1999). Genetic Monogamy in Long-Eared Owls. *The Condor*, *101*(4), 854–859. https://doi.org/10.2307/1370075

Marshall, R. C., Buchanan, K. L., & Catchpole, C. K. (2007). Song and female choice for extrapair copulations in the sedge warbler, Acrocephalus schoenobaenus. *Animal Behaviour*, *73*(4), 629–635. https://doi.org/10.1016/j.anbehav.2006.06.011

Martins, J. C., & Vasconcelos, V. M. (2009). Microcystin dynamics in aquatic organisms. *Journal of Toxicology and Environmental Health, Part B*, *12*(1), 65–82. https://doi.org/10.1080/10937400802545151

Masello, J. F., Sramkova, A., Quillfeldt, P., Epplen, J. T., & Lubjuhn, T. (2002). Genetic monogamy in burrowing parrots *Cyanoliseus patagonus* ? *Journal of Avian Biology*, *33*(1), 99–103. https://doi.org/10.1034/j.1600-048X.2002.330116.x

McLean, I. G., Kayes, S. D., Murie, J. O., Davis, L. S., & Lambert, D. M. (2000). Genetic monogamy mirrors social monogamy in the Fiordland crested penguin. *New Zealand Journal of Zoology*, *27*(4), 311–316. https://doi.org/10.1080/03014223.2000.9518240

Mee, A., Whitfield, D. P., Thompson, D. B. A., & Burke, T. (2004). Extrapair paternity in the common sandpiper, Actitis hypoleucos, revealed by DNA fingerprinting. *Animal Behaviour*, *67*(2), 333–342. https://doi.org/10.1016/j.anbehav.2003.02.007

Meek, S. B., Robertson, R. J., & Boag, P. T. (1994). Extrapair paternity and intraspecific brood parasitism in eastern bluebirds revealed by DNA fingerprinting. *The Auk*, *111*(3), 739–744.

Mennill, D. J., Ramsay, S. M., Boag, P. T., & Ratcliffe, L. M. (2004). Patterns of extrapair mating in relation to male dominance status and female nest placement in black-capped chickadees. *Behavioral Ecology*, *15*(5), 757–765. https://doi.org/10.1093/beheco/arh076

Michalek, K., & Winkler, H. (2001). Parental Care and Parentage in Monogamous Great Spotted Woodpeckers (Picoides major. And Middle Spotted Woodpeckers (Picoides medius). *Behaviour*, *138*(10), 1259–1285. https://doi.org/10.1163/15685390152822210

Millar, C. D., Anthony, I., Lambert, D. M., Stapleton, P. M., Bergmann, C. C., Bellamy, A. R., & Young, E. C. (1994). Patterns of reproductive success determined by DNA fingerprinting in a communally breeding oceanic bird. *Biological Journal of the Linnean Society*, *52*(1), 31–48. https://doi.org/10.1006/bijl.1994.1037

Millar, C. D., Lambert, D. M., & Young, E. C. (1997). Minisatellite DNA Detects Sex, Parentage, and Adoption in the South Polar Skua. *J. Hered*, *88*, 235–238.

Mingju, E., Gong, Y., Yu, J., Zhang, S., Qianxi, F., Jiang, Y., & Wang, H. (2017). Low level of extra-pair paternity between nearest neighbors results from female preference for high-quality males in the yellow-rumped flycatcher (Ficedula zanthopygia). *PLOS ONE*, *12*(3), e0172713. https://doi.org/10.1371/journal.pone.0172713

Minias, P., Wojczulanis-Jakubas, K., Rutkowski, R., Kaczmarek, K., & Janiszewski, T. (2016). Spatial patterns of extra-pair paternity in a waterbird colony: Separating the effects of nesting density and nest site location. *Behavioral Ecology and Sociobiology*, *70*(3), 369–376. https://doi.org/10.1007/s00265-015-2056-0

Miño, C. I., Pollet, I. L., Bishop, C. A., & Russello, M. A. (2011). Genetic mating system and population history of the endangered Western Yellow-breasted Chat (*Icteria virens auricollis*) in British Columbia, Canada. *Canadian Journal of Zoology*, *89*(10), 881–891. https://doi.org/10.1139/z11-061

Miño, C. I., Russello, M. A., Mussi Gonçalves, P. F., & Del Lama, S. N. (2011). Reconstructing genetic mating systems in the absence of parental information in colonially breeding waterbirds. *BMC Evolutionary Biology*, *11*(1), 196. https://doi.org/10.1186/1471-2148-11-196

Mitrus, J., Mitrus, C., Rutkowski, R., & Sikora, M. (2014). Extra-pair Paternity in Relation to Age of the Red-breasted Flycatcher *Ficedula Parva* males. *Avian Biology Research*, *7*(2), 111–116. https://doi.org/10.3184/175815514X13948188185179

Møller, A. P., & Tegelström, H. (1997). Extra-pair paternity and tail ornamentation in the barn swallow Hirundo rustica. *Behavioral Ecology and Sociobiology*, *41*(5), 353–360. https://doi.org/10.1007/s002650050395

Moreno, J., Boto, L., Fargallo, J. A., De León, A., & Potti, J. (2000). Absence of extra-pair fertilisations in the Chinstrap Penguin Pygoscelis antarctica. *Journal of Avian Biology*, *31*(4), 580–583. https://doi.org/10.1034/j.1600-048X.2000.1310418.x

Moreno, J., Martínez, J. G., González-Braojos, S., Ruiz-de-Castañeda, R., Cantarero, A., & Sánchez-Tójar, A. (2013). Extra-pair matings, context-dependence and offspring quality: A brood manipulation experiment in pied flycatchers. *Behaviour*, *150*(3–4), 359–380. https://doi.org/10.1163/1568539X-00003056

Morton, E. S., Stutchbury, B. J. M., Howlett, J. S., & Piper, W. H. (1998). Genetic monogamy in blue-headed vireos and a comparison with a sympatric vireo with extrapair paternity. *Behavioral Ecology*, *9*(5), 515–524. https://doi.org/10.1093/beheco/9.5.515

Mota, P. G., & Hoi-Leitner, M. (2003). Intense extrapair behaviour in a semicolonial passerine does not result in extrapair fertilizations. *Animal Behaviour*, *66*(6), 1019–1026. https://doi.org/10.1006/anbe.2002.2285

Muck, C., Kempenaers, B., Kuhn, S., Valcu, M., & Goymann, W. (2009). Paternity in the classical polyandrous black coucal (Centropus grillii)—A cuckoo accepting cuckoldry? *Behavioral Ecology*, *20*(6), 1185–1193. https://doi.org/10.1093/beheco/arp118

Müller, W., Epplen, J. T., & Lubjuhn, T. (2001). Genetic paternity analyses in Little Owls (Athene noctua): Does the high rate of paternal care select against extra-pair young? *Journal Für Ornithologie*, *142*(2), 195. https://doi.org/10.1046/j.1439-0361.2001.00069.x

Negro, J. J., Villarroel, M., Tella, J. L., Kuhnlein, U., Hiraldo, F., Donazar, J. A., & Bird, D. M. (1996). DNA fingerprinting reveals a low incidence of extra-pair fertilizations in the lesser kestrel. *Animal Behaviour*, *51*(4), 935–943. https://doi.org/10.1006/anbe.1996.0097

Nelson-Flower, M. J., Hockey, P. A. R., O’Ryan, C., Raihani, N. J., du Plessis, M. A., & Ridley, A. R. (2011). Monogamous dominant pairs monopolize reproduction in the cooperatively breeding pied babbler. *Behavioral Ecology*, *22*(3), 559–565. https://doi.org/10.1093/beheco/arr018

Neto, J., Hansson, B., & Hasselquist, D. (2010). Low frequency of extra-pair paternity in Savi’s Warblers (Locustella luscinioides). *Behaviour*, *147*(11), 1413–1429. https://doi.org/10.1163/000579510X517217

O’Brien, E. L., & Dawson, R. D. (2007). Context-dependent genetic benefits of extra-pair mate choice in a socially monogamous passerine. *Behavioral Ecology and Sociobiology*, *61*(5), 775–782. https://doi.org/10.1007/s00265-006-0308-8

Øigarden, T., Borge, T., & Lifjeld, J. T. (2010). Extrapair paternity and genetic diversity: The white-throated dipper Cinclus cinclus. *Journal of Avian Biology*, *41*(3), 248–257. https://doi.org/10.1111/j.1600-048X.2009.04847.x

Orell, M., Rytkönen, S., Launonen, V., Welling, P., Kumpulainen, K., Koivula, K., & Bachmann, L. (1997). Low frequency extra-pair paternity in the Willow Tit Parus montanus as revealed by DNA fingerprinting. *Ibis*, *139*(3), 562–566.

Overeem, K. R., Gabriel, P. O., Zirpoli, J. A., & Black, J. M. (2014). Steller Sex: Infidelity and Sexual Selection in a Social Corvid (Cyanocitta stelleri). *PLoS ONE*, *9*(8), e105257. https://doi.org/10.1371/journal.pone.0105257

Owens, I. P. F., Dixon, A., Burke, T., & Thompson, D. B. A. (1995). Strategic paternity assurance in the sex-role reversed Eurasian dotterel (*Charadrius morinellus*): Behavioral and genetic evidence. *Behavioral Ecology*, *6*(1), 14–21. https://doi.org/10.1093/beheco/6.1.14

Pechacek, P., Michalek, K. G., Winkler, H., & Blomqvist, D. (2005). Monogamy with exceptions: Social and genetic mating system in a bird species with high paternal investment. *Behaviour*, *142*(8), 1093–1114. https://doi.org/10.1163/156853905774405281

Petren, K., Grant, B. R., & Grant, P. R. (1999). Low Extrapair Paternity in the Cactus Finch (Geospiza scandens). *The Auk*, *116*(1), 252–256. https://doi.org/10.2307/4089475

Pierce, E. P., & Lifjeld, J. T. (1998). High Paternity without Paternity-Assurance Behavior in the Purple Sandpiper, a Species with High Paternal Investment. *The Auk*, *115*(3), 602–612. https://doi.org/10.2307/4089409

Pilastro, A., Pezzo, F., Olmastroni, S., Callegarin, C., Corsolini, S., & Focardi, S. (2001). Extrapair paternity in the Adélie Penguin Pygoscelis adeliae. *Ibis*, *143*, 681–684.

Pinxten, R., Hanotte, O., Eens, M., Verheyen, R. F., Dhondt, A. A., & Burke, T. (1993). Extra-pair paternity and intraspecific brood parasitism in the European starling, Sturnus vulgaris: Evidence from DNA fingerprinting. *Animal Behaviour*, *45*(4), 795–809. https://doi.org/10.1006/anbe.1993.1093

Piper, W. H., Evers, D. C., Meyer, M. W., Tischler, K. B., Kaplan, J. D., & Fleischer, R. C. (1997). Genetic monogamy in the common loon (Gavia immer). *Behavioral Ecology and Sociobiology*, *41*(1), 25–31. https://doi.org/10.1007/s002650050360

Põldmaa, T., Montgomerie, R., & Boag, P. (1995). Mating system of the cooperatively breeding noisy miner Manorina melanocephala, as revealed by DNA profiling. *Behavioral Ecology and Sociobiology*, *37*(2), 137–143. https://doi.org/10.1007/BF00164159

Pravosudova, E. V., Parker, P. G., & Gaunt, A. S. (2002). Genetic Evidence for Extrapair Paternity in the Tufted Titmouse. *The Wilson Bulletin*, *114*(2), 279–281. https://doi.org/10.1676/0043-5643(2002)114[0279:GEFEPI]2.0.CO;2

Quinn, J. S., Woolfenden, G. E., Fitzpatrick, J. W., & White, B. N. (1999). Multi-locus DNA fingerprinting supports genetic monogamy in Florida scrub-jays. *Behavioral Ecology and Sociobiology*, *45*(1), 1–10. https://doi.org/10.1007/s002650050534

Rabenold, P. P., Rabenold, K. N., Piper, W. H., Haydock, J., & Zack, S. W. (1990). Shared paternity revealed by genetic analysis in cooperatively breeding tropical wrens. *Nature*, *348*(6301), 538–540. https://doi.org/10.1038/348538a0

Reneerkens, J., van Veelen, P., van der Velde, M., Luttikhuizen, P., & Piersma, T. (2014). Within-population variation in mating system and parental care patterns in the Sanderling (*Calidris alba*) in northeast Greenland. *The Auk*, *131*(2), 235–247. https://doi.org/10.1642/AUK-13-247.1

Ribeiro, Â. M., Lloyd, P., Feldheim, K. A., & Bowie, R. C. K. (2012). Microgeographic socio-genetic structure of an African cooperative breeding passerine revealed: Integrating behavioural and genetic data. *Molecular Ecology*, *21*(3), 662–672. https://doi.org/10.1111/j.1365-294X.2011.05236.x

Riley, H. T., Bryant, D. M., Carter, R. E., & Parkin, D. T. (1995). Extra-pair fertilizations and paternity defence in house martins, Delichon urbica. *Animal Behaviour*, *49*(2), 495–509. https://doi.org/10.1006/anbe.1995.0065

Ritchison, G., Klatt, P. H., & Westneat, D. F. (1994). Mate Guarding and Extra-Pair Paternity in Northern Cardinals. *The Condor*, *96*(4), 1055–1063. https://doi.org/10.2307/1369114

Robertson, B. C., Degnan, S. M., Kikkawa, J., & Moritz, C. C. (2001). Genetic monogamy in the absence of paternity guards: The Capricorn silvereye, Zosterops lateralis chlorocephalus, on Heron Island. *Behavioral Ecology*, *12*(6), 666–673. https://doi.org/10.1093/beheco/12.6.666

Roeder, D. V., Husak, M. S., Murphy, M. T., & Patten, M. A. (2019). Size, ornamentation, and flight feather morphology promote within-pair paternity in a sexually dimorphic passerine. *Behavioral Ecology and Sociobiology*, *73*(7), 90. https://doi.org/10.1007/s00265-019-2704-x

Rosenfield, R. N., Sonsthagen, S. A., Stout, W. E., & Talbot, S. L. (2015). High frequency of extra-pair paternity in an urban population of Cooper’s Hawks. *Journal of Field Ornithology*, *86*(2), 144–152. https://doi.org/10.1111/jofo.12097

Rudnick, J. A., Katzner, T. E., Bragin, E. A., Rhodes, O. E., & Dewoody, J. A. (2005). Using naturally shed feathers for individual identification, genetic parentage analyses, and population monitoring in an endangered Eastern imperial eagle (Aquila heliaca) population from Kazakhstan. *Molecular Ecology*, *14*(10), 2959–2967. https://doi.org/10.1111/j.1365-294X.2005.02641.x

Rutkowski, R., Krupiński, D., Kitowski, I., Popović, D., Gryczyńska, A., Molak, M., Dulisz, B., Poprach, K., Müller, S., Müller, R., & Gierach, K.-D. (2015). Genetic structure and diversity of breeding Montagu’s harrier (Circus pygargus) in Europe. *European Journal of Wildlife Research*, *61*(5), 691–701. https://doi.org/10.1007/s10344-015-0943-3

Saladin, V., Ritschard, M., Roulin, A., Bize, P., & Richner, H. (2007). Analysis of genetic parentage in the tawny owl (Strix aluco) reveals extra-pair paternity is low. *Journal of Ornithology*, *148*(1), 113–116. https://doi.org/10.1007/s10336-006-0109-x

Saracura, V., Macedo, R. H., & Blomqvist, D. (2008). Genetic parentage and variable social structure in breeding southern lapwings. *The Condor*, *110*(3), 554–558. https://doi.org/10.1525/cond.2008.8477

Schamel, D., Tracy, D. M., Lank, D. B., & Westneat, D. F. (2004). Mate guarding, copulation strategies and paternity in the sex-role reversed, socially polyandrous red-necked phalarope Phalaropus lobatus. *Behavioral Ecology and Sociobiology*, *57*(2), 110–118. https://doi.org/10.1007/s00265-004-0825-2

Schwartz, M. K., Boness, D. J., Schaeff, C. M., Majluf, P., Perry, E. A., & Fleischer, R. C. (1999). Female-solicited extrapair matings in Humboldt penguins fail to produce extrapair fertilizations. *Behavioral Ecology*, *10*(3), 242–250. https://doi.org/10.1093/beheco/10.3.242

Seddon, N., Amos, W., Adcock, G., Johnson, P., Kraaijeveld, K., Kraaijeveld-Smit, F. J. L., Lee, W., Senapathi, G. D., Mulder, R. A., & Tobias, J. A. (2005). Mating system, philopatry and patterns of kinship in the cooperatively breeding subdesert mesite Monias benschi. *Molecular Ecology*, *14*(11), 3573–3583. https://doi.org/10.1111/j.1365-294X.2005.02675.x

Segelbacher, G., Kabisch, D., Stauss, M., & Tomiuk, J. (2005). Extra-pair young despite strong pair bonds in the European Nuthatch (Sitta europaea). *Journal of Ornithology*, *146*(2), 99–102. https://doi.org/10.1007/s10336-004-0062-5

Semple, K., Wayne, R. K., & Gibson, R. M. (2001). Microsatellite analysis of female mating behaviour in lek-breeding sage grouse. *Molecular Ecology*, *10*(8), 2043–2048. https://doi.org/10.1046/j.0962-1083.2001.01348.x

Shealer, D. A., Devbhandari, S., & Garcia-Mendoza, M. G. (2014). Evidence for Genetic Monogamy But Low Mate Retention in the North American Black Tern (*Chlidonias niger surinamensis*). *Waterbirds*, *37*(2), 129–135. https://doi.org/10.1675/063.037.0201

Smith, H. G., Montgomerie, R., Poldman, T., White, B. N., & Boag, P. T. (1991). DNA fingerprinting reveals relation between tail ornaments and cuckoldry in barn swallows, Hirundo rustica. *Behavioral Ecology*, *2*(1), 90–98. https://doi.org/10.1093/beheco/2.1.90

Smith, H. G., & von Schantz, T. (1993). Extra-Pair Paternity in the European Starling: The Effect of Polygyny. *The Condor*, *95*(4), 1006–1015. https://doi.org/10.2307/1369436

Smith, S. B., McKay, J. E., Murphy, M. T., & Duffield, D. A. (2016). Spatial patterns of extra‐pair paternity for spotted towhees *Pipilo maculatus* in urban parks. *Journal of Avian Biology*, *47*(6), 815–823. https://doi.org/10.1111/jav.00931

Soukup, S. S., & Thompson, C. F. (1997). Social mating system affects the frequency of extra-pair paternity in house wrens. *Animal Behaviour*, *54*(5), 1089–1105. https://doi.org/10.1006/anbe.1997.0556

Sousa, B. F., & Westneat, D. F. (2013). Positive association between social and extra-pair mating in a polygynous songbird, the dickcissel (Spiza americana). *Behavioral Ecology and Sociobiology*, *67*(2), 243–255. https://doi.org/10.1007/s00265-012-1444-y

St Clair, C. C., Waas, J. R., St Clair, R. C., & Boag, P. T. (1995). Unfit mothers? Maternal infanticide in royal penguins. *Animal Behaviour*, *50*(5), 1177–1185. https://doi.org/10.1016/0003-3472(95)80034-4

Stanback, M., Richardson, D. S., Boix-Hinzen, C., & Mendelsohn, J. (2002). Genetic monogamy in Monteiro’s hornbill, Tockus monteiri. *Animal Behaviour*, *63*(4), 787–793. https://doi.org/10.1006/anbe.2001.1975

Stapleton, M. K., Kleven, O., Lifjeld, J. T., & Robertson, R. J. (2007). Female tree swallows (Tachycineta bicolor) increase offspring heterozygosity through extrapair mating. *Behavioral Ecology and Sociobiology*, *61*(11), 1725–1733. https://doi.org/10.1007/s00265-007-0404-4

Stewart, S. L. M., Westneat, D. F., & Ritchison, G. (2010). Extra-pair paternity in eastern bluebirds: Effects of manipulated density and natural patterns of breeding synchrony. *Behavioral Ecology and Sociobiology*, *64*(3), 463–473. https://doi.org/10.1007/s00265-009-0862-y

Strohbach, S., Curio, E., Bathen, A., Epplen, J., & Lubjuhn, T. (1998). Extrapair paternity in the great tit (*Parus major*): A test of the “good genes” hypothesis. *Behavioral Ecology*, *9*(4), 388–396. https://doi.org/10.1093/beheco/9.4.388

Sundberg, J., & Dixon, A. (1996). Old, colourful male yellowhammers,Emberiza citrinella, benefit from extra-pair copulations. *Animal Behaviour*, *52*(1), 113–122. https://doi.org/10.1006/anbe.1996.0157

Swatschek, I., Ristow, D., Scharlau, W., Wink, C., & Wink, M. (1993). Populationsgenetik und Vaterschaftanalyse beim Elernorenfalken (Falco elenorae). *J. Ornithol*, *134*, 137–143.

Tarwater, C. E., Brawn, J. D., & Maddox, J. D. (2013). Low extrapair paternity observed in a tropical bird despite ample opportunities for extrapair mating. *The Auk*, *130*(4), 733–741. https://doi.org/10.1525/auk.2013.13117

Taylor, S. S., Boessenkool, S., & Jamieson, I. G. (2008). Genetic monogamy in two long-lived New Zealand passerines. *Journal of Avian Biology*, *39*(5), 579–583. https://doi.org/10.1111/j.0908-8857.2008.04331.x

Triggs, S., Williams, M., Marshall, S., & Chambers, G. (1991). Genetic relationships within a population of Blue Duck Hymenolaimus malacorhynchos. *Wildfowl*, *42*, 87–93.

Václav, R., & Hoi, H. (2007). Experimental manipulation of timing of breeding suggests laying order instead of breeding synchrony affects extra-pair paternity in house sparrows. *Journal of Ornithology*, *148*(4), 395–400. https://doi.org/10.1007/s10336-007-0143-3

Valera, F., Hoi, H., & Krištín, A. (2003). Male shrikes punish unfaithful females. *Behavioral Ecology*, *14*(3), 403–408. https://doi.org/10.1093/beheco/14.3.403

Vallender, R., Friesen, V. L., & Robertson, R. J. (2007). Paternity and performance of golden-winged warblers (Vermivora chrysoptera) and golden-winged X blue-winged warbler (V. pinus) hybrids at the leading edge of a hybrid zone. *Behavioral Ecology and Sociobiology*, *61*(12), 1797–1807. https://doi.org/10.1007/s00265-007-0413-3

van den Heuvel, I. M., Cherry, M. I., & Klump, G. M. (2014). Crimson-breasted Shrike females with extra pair offspring contributed more to duets. *Behavioral Ecology and Sociobiology*, *68*(8), 1245–1252. https://doi.org/10.1007/s00265-014-1735-6

van Dongen, W. F. D., & Mulder, R. A. (2009). Multiple ornamentation, female breeding synchrony, and extra-pair mating success of golden whistlers (Pachycephala pectoralis). *Journal of Ornithology*, *150*(3), 607–620. https://doi.org/10.1007/s10336-009-0371-9

van Oers, K., Drent, P. J., Dingemanse, N. J., & Kempenaers, B. (2008). Personality is associated with extrapair paternity in great tits, Parus major. *Animal Behaviour*, *76*(3), 555–563. https://doi.org/10.1016/j.anbehav.2008.03.011

Veiga, J. P., & Boto, L. (2000). Low frequency of extra-pair fertilisations in House Sparrows breeding at high density. *Journal of Avian Biology*, *31*(2), 237–244. https://doi.org/10.1034/j.1600-048X.2000.310215.x

Voigt, C., Leitner, S., & Gahr, M. (2003). Mate fidelity in a population of Island Canaries (Serinus canaria) in the Madeiran Archipelago. *Journal Für Ornithologie*, *144*, 86–92. https://doi.org/10.1046/j.1439-0361.2003.02047.x

Wallander, J., Blomqvist, D., & Lifjeld, J. T. (2001). Genetic and Social Monogamy—Does It Occur Without Mate Guarding in the Ringed Plover? *Ethology*, *107*(7), 561–572. https://doi.org/10.1046/j.1439-0310.2001.00695.x

Warkentin, I. G., D.-Curzon, A., Carter, R. E., Wetton, J. H., James, P. C., Oliphant, L. W., & Parkin, D. T. (1994). No evidence for extrapair fertilizations in the merlin revealed by DNA fingerprinting. *Molecular Ecology*, *3*(3), 229–234. https://doi.org/10.1111/j.1365-294X.1994.tb00056.x

Warrington, M. H., Rollins, L. A., Raihani, N. J., Russell, A. F., & Griffith, S. C. (2013). Genetic monogamy despite variable ecological conditions and social environment in the cooperatively breeding apostlebird. *Ecology and Evolution*, *3*(14), 4669–4682. https://doi.org/10.1002/ece3.844

Wiebe, K. L., & Kempenaers, B. (2009). The social and genetic mating system in flickers linked to partially reversed sex roles. *Behavioral Ecology*, *20*(2), 453–458. https://doi.org/10.1093/beheco/arn138

Wink, M., Becker, D., Tolkmitt, D., Knigge, V., Sauer-Gürth, H., & Staudter, H. (2011). Mating system, paternity and sex allocation in Eurasian Wrynecks (Jynx torquilla). *Journal of Ornithology*, *152*(4), 983–989. https://doi.org/10.1007/s10336-011-0684-3

Winkel, W., Winkel, D., & Lubjuhn, T. (2001). Vaterschaftsnachweise bei vier ungewöhnlich dicht benachbart brütenden Kohlmeisen-Paaren (Parus major). *Journal für Ornithologie*, *142*(4), 429–432. https://doi.org/10.1007/BF01651341

Winterbottom, M., Burke, T., & Birkhead, T. (2001). The phalloid organ, orgasm and sperm competition in a polygynandrous bird: The red-billed buffalo weaver (Bubalornis niger). *Behavioral Ecology and Sociobiology*, *50*(5), 474–482. https://doi.org/10.1007/s002650100384

Woolaver, L. G., Nichols, R. K., Morton, E. S., & Stutchbury, B. J. M. (2013). Social and genetic mating system of Ridgway’s hawk (*Buteo ridgwayi*), an endemic raptor on Hispaniola. *Journal of Tropical Ecology*, *29*(6), 531–540. https://doi.org/10.1017/S0266467413000655

Woolfenden, B. E., Stutchbury, B. J. M., & Morton, E. S. (2005). Male Acadian flycatchers, Empidonax virescens, obtain extrapair fertilizations with distant females. *Animal Behaviour*, *69*(4), 921–929. https://doi.org/10.1016/j.anbehav.2004.06.030

Woxvold, I. A., & Mulder, R. A. (2008). Mixed mating strategies in cooperatively breeding apostlebirds Struthidea cinerea. *Journal of Avian Biology*, *39*(1), 50–56. https://doi.org/10.1111/j.0908-8857.2008.04102.x

Yamagishi, S., Nishiumi, I., & Shimoda, C. (1992). Extrapair Fertilization in Monogamous Bull-Headed Shrikes Revealed by DNA Fingerprinting. *The Auk*, *109*(4), 711–721. https://doi.org/10.2307/4088147

Yezerinac, S., Lanctot, R. B., Sage, G. K., & Talbot, S. L. (2013). Social and Genetic Mating System of the American Golden-Plover. *The Condor*, *115*(4), 808–815. https://doi.org/10.1525/cond.2013.120081

Zeng, L., Rotenberry, J. T., Zuk, M., Pratt, T. K., & Zhang, Z. (2016). Social behavior and cooperative breeding in a precocial species: The Kalij Pheasant (*Lophura leucomelanos*) in Hawaii. *The Auk*, *133*(4), 747–760. https://doi.org/10.1642/AUK-15-227.1

Zharikov, Y., & Nol, E. (2000). Copulation Behavior, Mate Guarding, and Paternity in the Semipalmated Plover. *The Condor*, *102*(1), 231–235. https://doi.org/10.1093/condor/102.1.231
