# Supplementary material for: How much multiple paternity should we expect? A study of birds and contrast with mammals
Source: Ecol Evol. 2024 Mar 1;14(3):e11054. doi: 10.1002/ece3.11054 (PMC10905237; doi:10.1002/ece3.11054)
Supplement: Supplementary file 3 — Appendix B. [file ECE3-14-e11054-s002.docx]

**Appendix B – Data References for Mammals**

| **Species** | **Reference** |
| --- | --- |
| African wild dog, Lycaon pictus | Girman et al., 1997 |
| Agile antechinus, Antechinus agilis | Kraaijeveld-Smit et al., 2002 |
| Alpine marmot, Marmota marmota | Cohas et al., 2006 |
| American black bear, Ursus americanus | Onorato et al., 2004 |
| American mink, Neovison vison | Yamaguchi et al., 2004 |
| Arctic fox, Vulpes lagopus | Carmichael et al., 2007 |
| Asian lesser white-toothed shrew, Crocidura shantungensis | Lin et al., 2009 |
| Big brown bat, Eptesicus fuscus | Vonhof et al., 2005 |
| Big-eared woodrat, Neotoma macrotis | Matocq, 2004 |
| Brandt's vole, Lasiopodomys brandtii | Huo et al., 2010 |
| Brazilian guinea pig, Cavia aperea | Asher et al., 2008 |
| Brown bear, Ursus arctos | Bellemain et al., 2006 |
| Canyon mouse, Peromyscus crinitus | Shurtliff et al., 2005 |
| Cape dune mole rat, Bathyergus suillus | Bray et al., 2012 |
| Cheetah, Acinonyx jubatus | Gotelli, 2001 |
| Columbian ground squirrel, Urocitellus columbianus | Jones et al., 2012; Raveh, 2010 |
| Domestic cat, Felis catus | Natoli et al., 2007; Say et al., 1999 |
| Eastern chipmunk, Tamias straitus | Bergeron et al., 2011 |
| Eastern red bat, Lasiurus borealis | Ammerman et al., 2019 |
| Ermine, Mustela erminea | Holland & Gleeson, 2005 |
| Ethiopian wolf, Canis simensis | Randall et al., 2007 |
| Eurasian badger, Meles meles | Carpenter et al., 2004; Dugdale et al., 2007 |
| European edible dormouse, Glis glis | (Moska et al., 2021) |
| Gray fox, Urocyon cinereoargenteus | Weston Glenn et al., 2009 |
| Gray mouse lemur, Microcebus murinus | Eberle & Kappeler, 2004 |
| Grey red-backed vole, Myodes rufocanus | Ishibashi & Saitoh, 2008 |
| Guadeloupe racoon, Procyon lotor | Nielsen & Nielsen, 2007 |
| Honey possum, Tarsipes rostratus | Wooller et al., 2000 |
| House mouse, Mus musculus | Auclair et al., 2014; Dean et al., 2006 |
| Large treeshrew, Tupaia tana | Munshi-South, 2007 |
| Mouflon, Ovis aries | Pemberton et al., 1999 |
| Natal multimammate mouse, Mastomys natalensis | Kennis et al., 2008 |
| North American Beaver, Castor canadensis | Crawford et al., 2008 |
| Northern quoll, Dasyurus hallucatus | Chan et al., 2020 |
| Prairie vole, Microtus ochrogaster | Solomon et al., 2004 |
| Pronghorn, Antilocapra smericana | Carling et al., 2003 |
| Red fox, Vulpes vulpes | Baker et al., 2004 |
| Red squirrel, Tamiasciurus hudsonicus | Bonanno & Schulte-Hostedde, 2009; Lane et al., 2008 |
| Richardson's ground squirrel, Urocitellus richardsonii | Hare et al., 2004 |
| Roe deer, Capreolus capreolus | Vanpé et al., 2009 |
| Round-tailed ground squirrel, Xerospermophilus tereticaudus | Munroe & Koprowski, 2011 |
| Silvery mole rat, Heliophobius argenteocinereus | Patzenhauerová et al., 2010 |
| Snowshoe hare, Lepus americanus | Burton, 2002 |
| Spotted hyena, Crocuta crocuta | East et al., 2003; Engh, 2002 |
| Spotted-tailed quoll, Dasyurus maculatus | Glen et al., 2008 |
| Striped ﬁeld mouse, Apodemus agrarius | Baker et al., 1999; Bryja et al., 2008 |
| Striped hyena, Hyaena hyaena | Wagner et al., 2007 |
| Stuart's antechinus, Antechinus stuartii | Holleley et al., 2006 |
| Swamp antechinus, Antechinus minimus | Sale et al., 2013 |
| Tundra vole, Microtus oeconomus | Borkowska et al., 2009 |
| Ural ﬁeld mouse, Apodemus uralensis | Bryja et al., 2008 |
| Virginia opossum, Didelphis virginiana | Beasley et al., 2010 |
| Western European hedgehog, Erinaceus europaeus | Moran et al., 2009 |
| White-tailed deer, Odocoileus virginianus | Neuman et al., 2016; Sorin, 2004 |
| White-toothed shrew, Crocidura russula | Bouteiller & Perrin, 2000 |
| Wild boar, Sus scrofa | Delgado et al., 2008; Poteaux et al., 2009 |
| Wolverine, Gulo gulo | Hedmark et al., 2007 |
| Wood mouse, Apodemus sylvaticus | Baker et al., 1999; Bryja et al., 2008 |
| Woodchuck, Marmota monax | Maher & Duron, 2010 |
| Yellow ground squirrel, Spermophilus fulvus | (Batova et al., 2021) |
| Yellow-necked field mouse, Apodemus flavicollis | Bryja et al., 2008; Gryczyńska-Siemiątkowska et al., 2008 |

**References**

Ammerman, L. K., Lee, D. N., Jones, B. A., Holt, M. P., Harrison, S. J., & Decker, S. K. (2019). High Frequency of Multiple Paternity in Eastern Red Bats, Lasiurus borealis, Based on Microsatellite Analysis. *Journal of Heredity*, *110*(6), 675–683. https://doi.org/10.1093/jhered/esz044

Asher, M., Lippmann, T., Epplen, J. T., Kraus, C., Trillmich, F., & Sachser, N. (2008). Large males dominate: Ecology, social organization, and mating system of wild cavies, the ancestors of the guinea pig. *Behavioral Ecology and Sociobiology*, *62*(9), 1509–1521. https://doi.org/10.1007/s00265-008-0580-x

Auclair, Y., König, B., & Lindholm, A. K. (2014). Socially mediated polyandry: A new benefit of communal nesting in mammals. *Behavioral Ecology*, *25*(6), 1467–1473. https://doi.org/10.1093/beheco/aru143

Baker, P. J., Funk, S. M., Bruford, M. W., & Harris, S. (2004). Polygynandry in a red fox population: Implications for the evolution of group living in canids? *Behavioral Ecology*, *15*(5), 766–778. https://doi.org/10.1093/beheco/arh077

Baker, R. J., Makova, K. D., & Chesser, R. K. (1999). Microsatellites indicate a high frequency of multiple paternity in *Apodemus* (Rodentia). *Molecular Ecology*, *8*(1), 107–111. https://doi.org/10.1046/j.1365-294X.1999.00541.x

Batova, O. N., Vasilieva, N. A., Titov, S. V., Savinetskaya, L. E., & Tchabovsky, A. V. (2021). Female polyandry dilutes inbreeding in a solitary fast-living hibernator. *Behavioral Ecology and Sociobiology*, *75*(10), 145. https://doi.org/10.1007/s00265-021-03086-1

Beasley, J. C., Beatty, W. S., Olson, Z. H., & Rhodes, O. E. (2010). A Genetic Analysis of the Virginia Opossum Mating System: Evidence of Multiple Paternity in a Highly Fragmented Landscape. *Journal of Heredity*, *101*(3), 368–373. https://doi.org/10.1093/jhered/esp114

Bellemain, E., Swenson, J. E., & Taberlet, P. (2006). Mating Strategies in Relation to Sexually Selected Infanticide in a Non-Social Carnivore: The Brown Bear. *Ethology*, *112*(3), 238–246. https://doi.org/10.1111/j.1439-0310.2006.01152.x

Bergeron, P., Réale, D., Humphries, M. M., & Garant, D. (2011). Evidence of multiple paternity and mate selection for inbreeding avoidance in wild eastern chipmunks. *Journal of Evolutionary Biology*, *24*(8), 1685–1694. https://doi.org/10.1111/j.1420-9101.2011.02294.x

Bonanno, V. L., & Schulte-Hostedde, A. I. (2009). Sperm competition and ejaculate investment in red squirrels (Tamiasciurus hudsonicus). *Behavioral Ecology and Sociobiology*, *63*(6), 835–846. https://doi.org/10.1007/s00265-009-0718-5

Borkowska, A., Borowski, Z., & Krysiuk, K. (2009). Multiple paternity in free-living root voles (Microtus oeconomus). *Behavioural Processes*, *82*(2), 211–213. https://doi.org/10.1016/j.beproc.2009.05.003

Bouteiller, C., & Perrin, N. (2000). Individual reproductive success and effective population size in the greater white–toothed shrew *Crocidura russula*. *Proceedings of the Royal Society of London. Series B: Biological Sciences*, *267*(1444), 701–705. https://doi.org/10.1098/rspb.2000.1059

Bray, T. C., Bloomer, P., O’Riain, M. J., & Bennett, N. C. (2012). How Attractive Is the Girl Next Door? An Assessment of Spatial Mate Acquisition and Paternity in the Solitary Cape Dune Mole-Rat, Bathyergus suillus. *PLoS ONE*, *7*(6), e39866. https://doi.org/10.1371/journal.pone.0039866

Bryja, J., Patzenhauerová, H., Albrecht, T., Mošanský, L., Stanko, M., & Stopka, P. (2008). Varying levels of female promiscuity in four Apodemus mice species. *Behavioral Ecology and Sociobiology*, *63*(2), 251–260. https://doi.org/10.1007/s00265-008-0656-7

Burton, C. (2002). Microsatellite analysis of multiple paternity and male reproductive success in the promiscuous snowshoe hare. *Canadian Journal of Zoology*, *80*(11), 1948–1956. https://doi.org/10.1139/z02-187

Carling, M. D., Wiseman, P. A., & Byers, J. A. (2003). Microsatellite analysis reveals multiple paternity in a population of wild pronghorn antelopes (Antilocapra americana). *Journal of Mammalogy*, *84*(4), 1237–1243. https://doi.org/10.1644/BRB-116

Carmichael, L. E., Szor, G., Berteaux, D., Giroux, M. A., Cameron, C., & Strobeck, C. (2007). Free love in the far north: Plural breeding and polyandry of arctic foxes (*Alopex lagopus*) on Bylot Island, Nunavut. *Canadian Journal of Zoology*, *85*(3), 338–343. https://doi.org/10.1139/Z07-014

Carpenter, P. J., Pope, L. C., Greig, C., Dawson, D. A., Rogers, L. M., Erven, K., Wilson, G. J., Delahay, R. J., Cheeseman, C. L., & Burke, T. (2004). Mating system of the Eurasian badger, Meles meles, in a high density population. *Molecular Ecology*, *14*(1), 273–284. https://doi.org/10.1111/j.1365-294X.2004.02401.x

Chan, R., Dunlop, J., & Spencer, P. B. S. (2020). Highly promiscuous paternity in mainland and island populations of the endangered Northern Quoll. *Journal of Zoology*, *310*(3), 210–220. https://doi.org/10.1111/jzo.12745

Cohas, A., Yoccoz, N. G., Da Silva, A., Goossens, B., & Allainé, D. (2006). Extra-pair paternity in the monogamous alpine marmot (Marmota marmota): The roles of social setting and female mate choice. *Behavioral Ecology and Sociobiology*, *59*(5), 597–605. https://doi.org/10.1007/s00265-005-0086-8

Crawford, J. C., Liu, Z., Nelson, T. A., Nielsen, C. K., & Bloomquist, C. K. (2008). Microsatellite analysis of mating and kinship in beavers (Castor canadensis). *Journal of Mammalogy*, *89*(3), 575–581. https://doi.org/10.1644/07-MAMM-A-251R1.1

Dean, M. D., Ardlie, K. G., & Nachman, M. W. (2006). The frequency of multiple paternity suggests that sperm competition is common in house mice ( *Mus domesticus* ). *Mol Ecol*, *15*, 4141–4151.

Delgado, R., Fernández-Llario, P., Azevedo, M., Beja-Pereira, A., & Santos, P. (2008). Paternity assessment in free-ranging wild boar (Sus scrofa) – Are littermates full-sibs? *Mammalian Biology*, *73*(3), 169–176. https://doi.org/10.1016/j.mambio.2007.07.008

Dugdale, H. L., Macdonald, D. W., Pope, L. C., & Burke, T. (2007). Polygynandry, extra-group paternity and multiple-paternity litters in European badger (*Meles meles*) social groups. *Molecular Ecology*, *16*(24), 5294–5306. https://doi.org/10.1111/j.1365-294X.2007.03571.x

East, M. L., Burke, T., Wilhelm, K., Greig, C., & Hofer, H. (2003). Sexual conflicts in spotted hyenas: Male and female mating tactics and their reproductive outcome with respect to age, social status and tenure. *Proceedings of the Royal Society of London. Series B: Biological Sciences*, *270*(1521), 1247–1254. https://doi.org/10.1098/rspb.2003.2363

Eberle, M., & Kappeler, P. M. (2004). Selected polyandry: Female choice and inter-sexual conflict in a small nocturnal solitary primate (Microcebus murinus). *Behavioral Ecology and Sociobiology*, *57*(1), 91–100. https://doi.org/10.1007/s00265-004-0823-4

Engh, A. L. (2002). Reproductive skew among males in a female-dominated mammalian society. *Behavioral Ecology*, *13*(2), 193–200. https://doi.org/10.1093/beheco/13.2.193

Girman, D. J., Mills, M. G. L., Geffen, E., & Wayne, R. K. (1997). A molecular genetic analysis of social structure, dispersal, and interpack relationships of the African wild dog (Lycaon pictus). *Behavioral Ecology and Sociobiology*, *40*(3), 187–198. https://doi.org/10.1007/s002650050332

Glen, A. S., Cardoso, M. J., Dickman, C. R., & Firestone, K. B. (2008). Who’s your daddy? Paternity testing reveals promiscuity and multiple paternity in the carnivorous marsupial Dasyurus maculatus (Marsupialia: Dasyuridae). *Biological Journal of the Linnean Society*, *96*(1), 1–7. https://doi.org/10.1111/j.1095-8312.2008.01094.x

Gotelli, N. J. (2001). Research frontiers in null model analysis. *Global Ecology and Biogeography*, *10*(4), 337–343. https://doi.org/10.1046/j.1466-822X.2001.00249.x

Gryczyńska-Siemiątkowska, A., Gortat, T., Kozakiewicz, A., Rutkowski, R., Pomorski, J., & Kozakiewicz, M. (2008). Multiple paternity in a wild population of the yellow-necked mouse Apodemus flavicollis. *Acta Theriologica*, *53*(3), 251–258. https://doi.org/10.1007/BF03193121

Hare, J. F., Todd, G., & Untereiner, W. A. (2004). Multiple Mating Results in Multiple Paternity in Richardson’s Ground Squirrels, Spermophilus richardsonii. *The Canadian Field-Naturalist*, *118*(1), 90. https://doi.org/10.22621/cfn.v118i1.888

Hedmark, E., Persson, J., Segerström, P., Landa, A., & Ellegren, H. (2007). Paternity and mating system in wolverines *Gulo gulo*. *Wildlife Biology*, *13*(sp2), 13–30. https://doi.org/10.2981/0909-6396(2007)13[13:PAMSIW]2.0.CO;2

Holland, O. J., & Gleeson, D. M. (2005). Genetic characterisation of blastocysts and the identification of an instance of multiple parternity in the stoat (Mustela erminea). *Conservation Genetics*, *6*(5), 855–858. https://doi.org/10.1007/s10592-005-9030-8

Holleley, C. E., Dickman, C. R., Crowther, M. S., & Oldroyd, B. P. (2006). Size breeds success: Multiple paternity, multivariate selection and male semelparity in a small marsupial, Antechinus stuartii. *Molecular Ecology*, *15*(11), 3439–3448. https://doi.org/10.1111/j.1365-294X.2006.03001.x

Huo, Y., Wan, X., Wolff, J. O., Wang, G., Thomas, S., Iglay, R. B., Leopold, B. D., & Liu, W. (2010). Multiple paternities increase genetic diversity of offspring in Brandt’s voles. *Behavioural Processes*, *84*(3), 745–749. https://doi.org/10.1016/j.beproc.2010.05.002

Ishibashi, Y., & Saitoh, T. (2008). Effect of Local Density of Males on the Occurrence of Multimale Mating in Gray-sided Voles (Myodes rufocanus). *Journal of Mammalogy*, *89*(2), 388–397. https://doi.org/10.1644/07-MAMM-A-036.1

Jones, P. H., Van Zant, J. L., & Dobson, F. S. (2012). Variation in reproductive success of male and female Columbian ground squirrels (*Urocitellus columbianus*). *Canadian Journal of Zoology*, *90*(6), 736–743. https://doi.org/10.1139/z2012-042

Kennis, J., Sluydts, V., Leirs, H., & van Hooft, W. F. P. (2008). Polyandry and polygyny in an African rodent pest species, Mastomys natalensis. *Mammalia*, *72*(3). https://doi.org/10.1515/MAMM.2008.025

Kraaijeveld-Smit, F., Ward, S., & Temple-Smith, P. (2002). Multiple paternity in a field population of a small carnivorous marsupial, the agile antechinus, Antechinus agilis. *Behavioral Ecology and Sociobiology*, *52*(1), 84–91. https://doi.org/10.1007/s00265-002-0485-z

Lane, J. E., Boutin, S., Gunn, M. R., Slate, J., & Coltman, D. W. (2008). Female multiple mating and paternity in free-ranging North American red squirrels. *Animal Behaviour*, *75*(6), 1927–1937. https://doi.org/10.1016/j.anbehav.2007.10.038

Lin, T.-T., You, E.-M., & Lin, Y. K. (2009). Social and Genetic Mating Systems of the Asian Lesser White-toothed Shrew, Crocidura shantungensis, in Taiwan. *Journal of Mammalogy*, *90*(6), 1370–1380. https://doi.org/10.1644/08-MAMM-A-346R1.1

Maher, C. R., & Duron, M. (2010). Mating system and paternity in woodchucks (*Marmota monax*). *Journal of Mammalogy*, *91*(3), 628–635. https://doi.org/10.1644/09-MAMM-A-324.1

Matocq, M. D. (2004). Reproductive success and effective population size in woodrats (Neotoma macrotis). *Molecular Ecology*, *13*(6), 1635–1642. https://doi.org/10.1111/j.1365-294X.2004.02173.x

Moran, S., Turner, P. D., & O’Reilly, C. (2009). Multiple paternity in the European hedgehog. *Journal of Zoology*, *278*(4), 349–353. https://doi.org/10.1111/j.1469-7998.2009.00583.x

Moska, M., Mucha, A., Wierzbicki, H., & Nowak, B. (2021). Edible dormouse (*Glis glis*) population study in south‐western Poland provides evidence of multiple paternity and communal nesting. *Journal of Zoology*, *314*(3), 194–202. https://doi.org/10.1111/jzo.12881

Munroe, K. E., & Koprowski, J. L. (2011). Sociality, Bateman’s gradients, and the polygynandrous genetic mating system of round-tailed ground squirrels (Xerospermophilus tereticaudus). *Behavioral Ecology and Sociobiology*, *65*(9), 1811–1824. https://doi.org/10.1007/s00265-011-1189-z

Munshi-South, J. (2007). Extra-pair paternity and the evolution of testis size in a behaviorally monogamous tropical mammal, the large treeshrew (Tupaia tana). *Behavioral Ecology and Sociobiology*, *62*(2), 201–212. https://doi.org/10.1007/s00265-007-0454-7

Natoli, E., Schmid, M., Say, L., & Pontier, D. (2007). Male Reproductive Success in a Social Group of Urban Feral Cats (Felis catus L.). *Ethology*, *113*(3), 283–289. https://doi.org/10.1111/j.1439-0310.2006.01320.x

Neuman, T. J., Newbolt, C. H., Ditchkoff, S. S., & Steury, T. D. (2016). Microsatellites reveal plasticity in reproductive success of white-tailed deer. *Journal of Mammalogy*, *97*(5), 1441–1450. https://doi.org/10.1093/jmammal/gyw087

Nielsen, C. L. R., & Nielsen, C. K. (2007). Multiple Paternity and Relatedness in Southern Illinois Raccoons (*Procyon lotor*). *Journal of Mammalogy*, *88*(2), 441–447. https://doi.org/10.1644/06-MAMM-A-126R2.1

Onorato, D. P., Hellgren, E. C., Van Den Bussche, R. A., & Skiles, Jr., J. R. (2004). Paternity and relatedness of American black bears recolonizing a desert montane island. *Canadian Journal of Zoology*, *82*(8), 1201–1210. https://doi.org/10.1139/z04-097

Patzenhauerová, H., Bryja, J., & Šumbera, R. (2010). Kinship structure and mating system in a solitary subterranean rodent, the silvery mole-rat. *Behavioral Ecology and Sociobiology*, *64*(5), 757–767. https://doi.org/10.1007/s00265-009-0893-4

Pemberton, J. M., Coltman, D. W., Smith, J. A., & Pilkington, J. G. (1999). Molecular analysis of a promiscuous, fluctuating mating system. *Biological Journal of the Linnean Society*, *68*(1–2), 289–301. https://doi.org/10.1111/j.1095-8312.1999.tb01170.x

Poteaux, C., Baubet, E., Kaminski, G., Brandt, S., Dobson, F. S., & Baudoin, C. (2009). Socio‐genetic structure and mating system of a wild boar population. *Journal of Zoology*, *278*(2), 116–125. https://doi.org/10.1111/j.1469-7998.2009.00553.x

Randall, D. A., Pollinger, J. P., Wayne, R. K., Tallents, L. A., Johnson, P. J., & Macdonald, D. W. (2007). Inbreeding is reduced by female-biased dispersal and mating behavior in Ethiopian wolves. *Behavioral Ecology*, *18*(3), 579–589. https://doi.org/10.1093/beheco/arm010

Raveh, S. (2010). Mating order and reproductive success in male Columbia ground squirrels (Urocitellus columbianus. *Behav Ecol*, *21*, 537–547.

Sale, M. G., Kraaijeveld-Smit, F. J. L., & Arnould, J. P. Y. (2013). Multiple paternity in the swamp antechinus (Antechinus minimus). *Australian Mammalogy*, *35*(2), 227. https://doi.org/10.1071/AM12039

Say, Ludovic., Pontier, Dominique., & Natoli, Eugenia. (1999). High variation in multiple paternity of domestic cats (Felis catus L.) in relation to environmental conditions. *Proceedings of the Royal Society of London. Series B: Biological Sciences*, *266*(1433), 2071–2074. https://doi.org/10.1098/rspb.1999.0889

Shurtliff, Q. R., Pearse, D. E., & Rogers, D. S. (2005). Parentage analysis of the canyon mouse (Peromyscus crinitus): Evidence for multiple paternity. *Journal of Mammalogy*, *86*(3), 531–540. https://doi.org/10.1644/1545-1542(2005)86[531:PAOTCM]2.0.CO;2

Solomon, N. G., Keane, B., Knoch, L. R., & Hogan, P. J. (2004). Multiple paternity in socially monogamous prairie voles (*Microtus ochrogaster*). *Canadian Journal of Zoology*, *82*(10), 1667–1671. https://doi.org/10.1139/z04-142

Sorin, A. B. (2004). Paternity assignment for white-tailed deer (Odocoileus virginianus): Mating across age classes and multiple paternity. *Journal of Mammalogy*, *85*(2), 356–362. https://doi.org/10.1644/1545-1542(2004)085<0356:PAFWDO>2.0.CO;2

Vanpé, C., Kjellander, P., Gaillard, J. M., Cosson, J. F., Galan, M., & Hewison, A. J. M. (2009). Multiple paternity occurs with low frequency in the territorial roe deer, Capreolus capreolus. *Biological Journal of the Linnean Society*, *97*(1), 128–139. https://doi.org/10.1111/j.1095-8312.2009.01196.x

Vonhof, M. J., Barber, D., Fenton, M. B., & Strobeck, C. (2005). A tale of two siblings: Multiple paternity in big brown bats (Eptesicus fuscus) demonstrated using microsatellite markers. *Molecular Ecology*, *15*(1), 241–247. https://doi.org/10.1111/j.1365-294X.2005.02801.x

Wagner, A. P., Creel, S., Frank, L. G., & Kalinowski, S. T. (2007). Patterns of relatedness and parentage in an asocial, polyandrous striped hyena population. *Molecular Ecology*, *16*(20), 4356–4369. https://doi.org/10.1111/j.1365-294X.2007.03470.x

Weston Glenn, J. L., Civitello, D. J., & Lance, S. L. (2009). Multiple paternity and kinship in the gray fox (Urocyon cinereoargenteus). *Mammalian Biology*, *74*(5), 394–402. https://doi.org/10.1016/j.mambio.2008.10.003

Wooller, R. D., Richardson, K. C., Garavanta, C. A. M., Saffer, V. M., & Bryant, K. A. (2000). Opportunistic breeding in the polyandrous honey possum, Tarsipes rostratus. *Australian Journal of Zoology*, *48*(6), 669. https://doi.org/10.1071/ZO00071

Yamaguchi, N., Sarno, R. J., Johnson, W. E., O’Brien, S. J., & Macdonald, D. W. (2004). Multiple Paternity and Reproductive Tactics of Free-Ranging American Minks, Mustela vison. *Journal of Mammalogy*. https://doi.org/10.1644/1383939
